# Detecting the Hidden Properties of Immunological Data and Predicting the Mortality Risks of Infectious Syndromes

**DOI:** 10.3389/fimmu.2016.00217

**Published:** 2016-06-10

**Authors:** S. Chatzipanagiotou, A. Ioannidis, E. Trikka-Graphakos, N. Charalampaki, C. Sereti, R. Piccinini, A. M. Higgins, T. Buranda, R. Durvasula, A. L. Hoogesteijn, G. P. Tegos, Ariel L. Rivas

**Affiliations:** ^1^Department of Biopathology and Clinical Microbiology, Aeginition Hospital, Medical School, National and Kapodistrian University of Athens, Athens, Greece; ^2^Department of Nursing, Faculty of Human Movement and Quality of Life Sciences, University of Peloponnese, Sparta, Greece; ^3^Department of Clinical Microbiology, “Thriasio” General Hospital, Magoula, Greece; ^4^Department of Veterinary Science and Public Health, University of Milan, Milan, Italy; ^5^Division of Infectious Diseases, Center for Global Health, School of Medicine, University of New Mexico, Albuquerque, NM, USA; ^6^Department of Pathology, School of Medicine, University of New Mexico, Albuquerque, NM, USA; ^7^Human Ecology Department, Cinvestav, Unidad Merida, Mexico; ^8^Torrey Pines Institute for Molecular Studies, Port St. Lucie, FL, USA; ^9^Department of Dermatology, Harvard Medical School, Boston, MA, USA; ^10^Wellman Center for Photomedicine, Massachusetts General Hospital, Boston, MA, USA

**Keywords:** complexity, immunomicrobial interactions, sepsis, immunosuppression, pattern recognition, visual

## Abstract

**Background:**

To extract more information, the properties of infectious disease data, including hidden relationships, could be considered. Here, blood leukocyte data were explored to elucidate whether hidden information, if uncovered, could forecast mortality.

**Methods:**

Three sets of individuals (*n* = 132) were investigated, from whom blood leukocyte profiles and microbial tests were conducted (i) cross-sectional analyses performed at admission (before bacteriological tests were completed) from two groups of hospital patients, randomly selected at different time periods, who met septic criteria [confirmed infection and at least three systemic inflammatory response syndrome (SIRS) criteria] but lacked chronic conditions (study I, *n* = 36; and study II, *n* = 69); (ii) a similar group, tested over 3 days (*n* = 7); and (iii) non-infected, SIRS-negative individuals, tested once (*n* = 20). The data were analyzed by (i) a method that creates complex data combinations, which, based on graphic patterns, partitions the data into subsets and (ii) an approach that does not partition the data. Admission data from SIRS+/infection+ patients were related to 30-day, in-hospital mortality.

**Results:**

The non-partitioning approach was not informative: in both study I and study II, the leukocyte data intervals of non-survivors and survivors overlapped. In contrast, the combinatorial method distinguished two subsets that, later, showed twofold (or larger) differences in mortality. While the two subsets did not differ in gender, age, microbial species, or antimicrobial resistance, they revealed different immune profiles. Non-infected, SIRS-negative individuals did not express the high-mortality profile. Longitudinal data from septic patients displayed the pattern associated with the highest mortality within the first 24 h post-admission. Suggesting inflammation coexisted with immunosuppression, one high-mortality sub-subset displayed high neutrophil/lymphocyte ratio values and low lymphocyte percents. A second high-mortality subset showed monocyte-mediated deficiencies. Numerous within- and between-subset comparisons revealed statistically significantly different immune profiles.

**Conclusion:**

While the analysis of non-partitioned data can result in information loss, complex (combinatorial) data structures can uncover hidden patterns, which guide data partitioning into subsets that differ in mortality rates and immune profiles. Such information can facilitate diagnostics, monitoring of disease dynamics, and evaluation of subset-specific, patient-specific therapies.

## Introduction

Awareness on the *properties* of immunological *data* may improve the study of infectious diseases. Here, infectious disease data-related properties are reviewed, and their desirable and undesirable consequences are considered in the process of developing a method meant to explore host–microbial interactions, which is subsequently pilot-tested.

Infectious disease data may exhibit at least five properties (i) *circularity*, (ii) *ambiguity*, (iii) *hidden* relationships, (iv) *dynamics*, and (v) *complexity*. Such features are associated with or may be influenced by *compositional*, *interdependent*, and *non-linear* relationships (1–15).

When antimicrobial immunological data are collected over time and analyzed in three-dimensional (3D) space, *circularity* is observed (1). Because circular data have no beginning and no end, classic statistics do not apply to such data (2–4).

Infectious disease data can be ambiguous: *numerically similar* data points may express *different biological conditions*. For instance, when lymphocyte (L), monocyte (M), or neutrophil (N) counts are analyzed, the same count can be generated by different percents (and *vice versa*), e.g., a count = 100 cells can consist of 60% N, 20% M, and 20% L (a healthy person), or 90% N, 5% M, and 5% L (a person with an inflammatory disorder).

Ambiguity may also be the result of *temporal changes* (dynamics) and/or *hidden* relationships (5). The analysis of *complexity* may uncover information usually unobserved (6–8).

Complexity involves four features (i) *emergence*, (ii) *irreducibility*, (iii) *unpredictability*, and (iv) *autonomy* (9–14). Emergence (or novelty) refers to patterns only detected when a complex (system-level) data structure is assembled. “Emergent” patterns may be alternative expressions of *hidden relationships* (5). Due to irreducibility and unpredictability, emergence cannot be reduced to or explained by any one variable, i.e., no “simple” and/or isolated variable can discriminate. Autonomy is associated with *non-linearity*: because causes and effects are not coupled, emergence (the effect) is numerically autonomous from the cause(s) (14).

Leukocyte data also exhibit *compositional* features. “*Composition*” is the term used since 1986 to describe systems characterized by three or more interacting classes –such as the three predominating cell types of the immune system (L, M, and N). Compositional data are not well described by counts (15). While percents and ratios have been proposed (16), they are not appropriate to analyze leukocyte data because the same ratio can be generated by different percents (and *vice versa*); e.g., an L/M ratio = 2 is generated both when L = 8%, M = 4%, and N = 88% (acute or recent inflammation) and also when L = 28%, M = 14%, and N = 58% (no inflammation).

To uncover hidden relationships –that is, to prevent ambiguity and detect “emergence” –the literature predicts that discrimination increases when the *levels of complexity* increase (17, 18). To increase and detect complexity, data *combinations* may be considered. Because the immune system is inherently *combinatorial*, approaches that measure combinations (interactions that involve two or more elements) have been proposed since 2000 (19, 20). Because lymphocytes and monocytes interact, at least, in antigen recognition processes (21, 22), they could be measured as interactions, e.g., using the L–M (or M–L) ratio. Because, over time, the same element can perform different functions –e.g., monocytes both promote and destroy neutrophils (23)–, dynamics should also be measured.

In addition, *dichotomization* should be avoided when interactions are assessed. The “cost of dichotomization” is the phrase used, since 1984, to describe the consequences of a numerical cutoff imposed on continuous data (e.g., leukocyte counts or percents). When a discontinuous (discrete) label is assigned to observations located above/below the cutoff (e.g., “infected”/“non-infected”), a substantial number of false (-positive and -negative) results will occur (24).

Therefore, some practices originated in non-biological fields (where biological complexity is not observed but data independence and linearity may be found) are not justified to study infectious disease data, which include non-linearly distributed data from interdependent leukocytes that, through interactions, perform different functions at different times (25–27).

Because linear classifiers, logistic regressions, as well as decision tree-based methods dichotomize, it is not surprising that such approaches are poorly predictive: when such approaches are analyzed in terms of “area under the curve” (AUC), they show values <75% (28, 29). Dichotomization-, AUC-based evaluations also share an assumption rarely observed in infections: their predictions are only valid when disease prevalence is 50% (30). Because leukocyte–microbial data interactions can include many levels of complexity and/or reveal *data ambiguity*, only methods that address such problems are desirable (31, 32). However, methods that depend on population metrics (those that utilize confidence intervals) cannot be used in personalized medicine –where patients may differ from the “average patient” (33, 34).

In contrast, approaches that do not assume the whole can be reduced to or predicted from any part (non-reductionist methods) may be adequate to explore infections, especially if they implement data partitioning and foster personalized practices. Such methods could unveil the information potentially embedded in infectious disease-related data (6, 19, 20, 35).

Here, a method that generates leukocyte data combinations (interactions) and investigates several levels of complexity was evaluated in infections. Two questions were asked (i) can blood leukocyte data possess hidden information? and (ii) if usually unobserved patterns are elicited, can complex data structures, derived from blood cells, forecast mortality?

## Materials and Methods

### Individuals

Two random samples of infected patients admitted to Greek hospitals in 2014, at different time frames, were analyzed. They had no history of chronic diseases but met at least three systemic inflammatory response syndrome (SIRS) criteria (36): body temperature >38°C, heart rate >90 beats/minute, tachypnea or hyperventilation (>20 breaths/minute or P_ACO2_ < 32 mm Hg at sea level), and white blood cell count ≥12,000 or ≤4000/μl. Such criteria characterize sepsis (36). Blood samples were taken at admission from 36 (study I) and 69 (study II) patients aged 31–87, and 30-day, in-hospital mortality was determined. In addition, 7 individuals meeting the same criteria were tested up to three times, daily, from the time of their admission; and 20 non-infected, SIRS-negative individuals were tested once. This study –conceived after patients died or were discharged– was approved by the Scientific Committee of the Thriasio Hospital, Magoula, Greece (protocol 57/16-02-2015) and the University of New Mexico, United States (protocol 13-463-T-HSC). Patient records were de-identified prior to analysis.

### Laboratory Methods

Human white blood cell counts and percentages, C-reactive protein (CRP), and conventional blood culture followed by susceptibility testing of the isolated microorganisms were performed. General blood examination was conducted with an automated hematology analyzer (Coulter LH 780 Analyzer, Beckman Coulter International SA, Nyon, Switzerland). Serum CPR was measured with an automated system (BN ProSpec System, Siemens AG, Erlangen, Germany). Blood cultures were performed with the automated Bactec 9249 instrument (Becton Dickinson, NJ, USA). The pathogens isolated from blood were identified and tested for their antimicrobial susceptibility with the automated microbiology system Phoenix 100 (Becton Dickinson, NJ, USA).

### Leukocyte Data Structures of Several Complexity Levels

A three-step method partitioned infectious disease data into subsets. It consisted of (i) *expansion* (a step that augments the number of data structures available for analysis, so that hidden patterns, if present, may be detected), (ii) pattern *recognition* [a step that removes non-informative structures but keeps those that display distinct patterns (e.g., data inflections) and, based on such patterns, partitions the data into subsets], and (iii) *statistical analysis of immune profiles* (a step that analyzes leukocyte data, within- and between-subsets).

To implement the first step, dimensionless indicators (DIs) were utilized (5, 37–39). The first step was performed with a proprietary algorithm that creates DIs (5, 37). DIs are the result of any combination of counts, percentages, ratios, or products derived from the primary variables, i.e., data on lymphocytes (L), macrophages/monocytes (M), and/or neutrophils (N). For instance, based on leukocyte percentages, a single number can summarize numerous relationships, e.g., those resulting from calculating [(M/L × N/M)/(N/L × L/M)] over [(M + L/N) × (L + N/M)/(N + M)/L × (M/N)]. Because the resulting number includes all cell types but is not limited to any known biological variable or dimension, the number is dimensionless. While each DI received an identifier (e.g., *AAA*), DIs had no biological definition.

Dimensionless indicators were used to build and measure many *levels* of *complexity*. For instance, in the DI described above, one level of complexity (level I) is measured by each ratio of the first element or “numerator” (M/L, N/M, N/L, and L/M). Two more interactions (of level II complexity) are captured by each product (M/L × N/M, N/L × L/M). Complexity level III is explored by the composite ratio of the numerator, which includes two products [(M/L × N/M)/(N/L × L/M)]. Because the second element (“denominator”) has the same structure, the number of interactions doubles. An additional interaction (complexity level IV) is generated when the numerator and the denominator are simultaneously analyzed. When three DIs are assessed in 3D space, the number of interactions increases three times and, in addition, one more interaction (level V complexity) is produced by the overall 3D relationship. Therefore, in this example, each 3D plot can measure at least (4 × 2 + 1 × 2 + 1 × 3 + 1) *58 interactions*, which cover *five levels of complexity*. However, because some elements include more than one cell type (e.g., M + L), the actual number of interactions and levels of complexity could be higher.

After data subsets were identified in the second step, DIs were not used anymore. Instead, in the third step, input leukocyte data were analyzed statistically. Statistical analyses were also conducted in the second step: when the exact point where 3D inflections occurred was unclear, an open-ended series of statistical tests was conducted, both before and after specific data points were assigned to different subsets. This process identified subsets that revealed the narrowest interval and were orthogonal to one another, detecting data subsets likely to differ at statistically significant levels when, later, leukocyte profiles were investigated. Together, this design (i) did not depend on numerical cutoffs (data partitioning was based on graphic patterns), (ii) did not focus on any one variable, but interactions, (iii) analyzed not one but several data structures, and (iv) conducted statistical tests after (not before) data subsets were distinguished.

When subsets differed statistically in immune profiles and mortality, internal validity was supported. When different populations showed similar patterns, external validity was likely.

### Statistical Analysis

The subset corresponding to each data point was determined by linking the identification number of each data point to the spatial patterns observed in 3D plots. Because DIs were hypothetical and showed non-linear relationships, they were biologically uninterpretable and statistically intractable (14). However, after data partitioning revealed orthogonality, statistical tests were justified (40), and leukocyte data were interpretable. Analyses of medians (Mann–Whitney test) or proportions (Chi-square test), as well as 3D plots, were conducted or created with a commercial package (Minitab Inc., State College, PA, USA). Tables S1–S7 in Supplementary Material report the data and statistical test results. The footnotes of Tables S1 and S2 in Supplementary Material describe a procedure that enables readers to reproduce the main findings.

## Results

**Figure 1:**
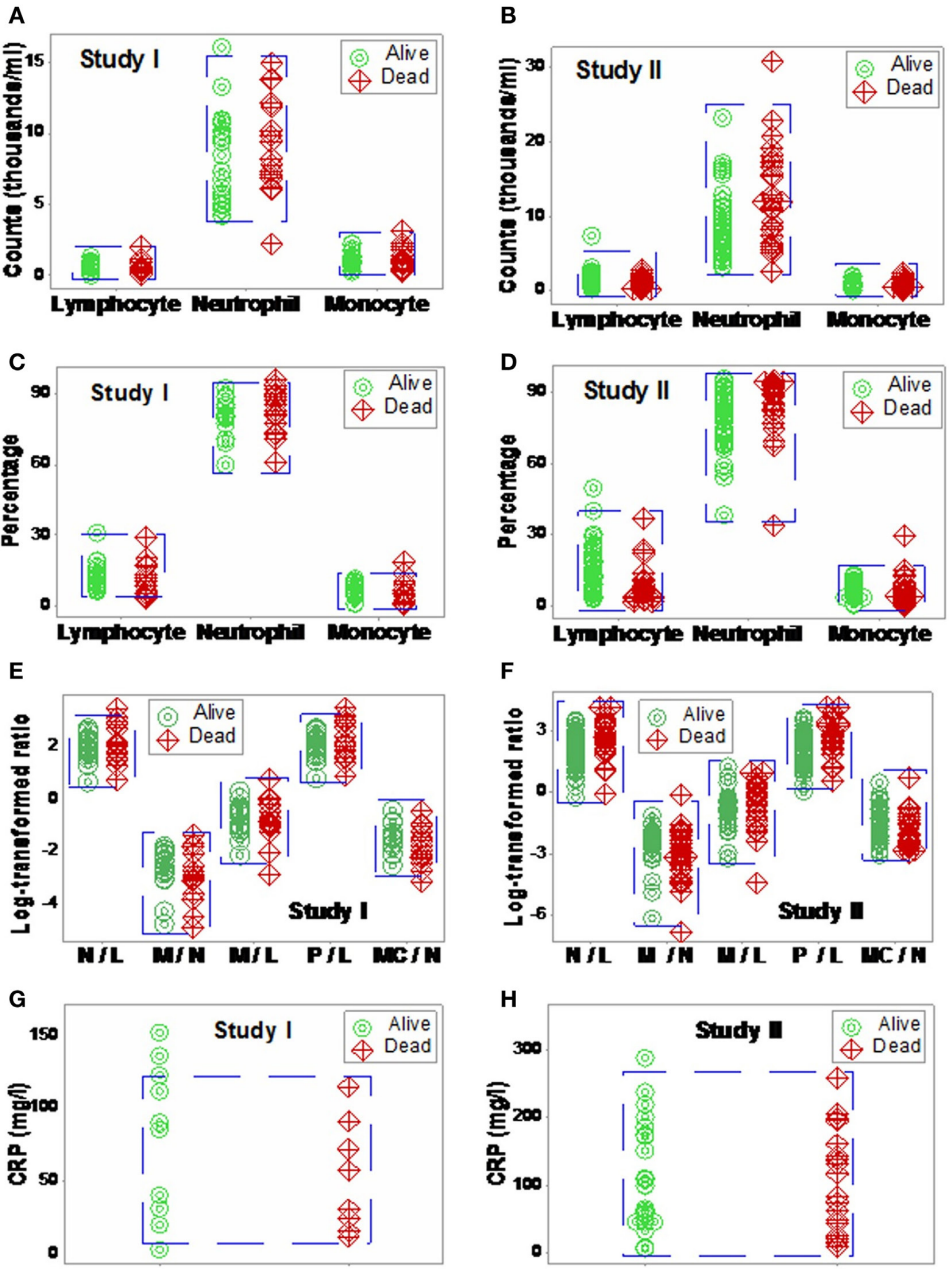
**Non-partitioning-based (non-combinatorial) analysis of leukocyte and biomarker data**. The outcomes reported within 30 days after SIRS+, infected individuals were admitted were not differentiated by input (blood leukocyte) data collected at admission. Both counts **(A,B)**, percentages **(C,D)**, and ratios of leukocyte cell types **(E,F)** as well as CRP concentrations **(G,H)** did not distinguish survivors from non-survivors: data overlapping was observed in both studies (blue boxes).

The analysis of separate variables (or variables that did not measure highly complex interactions) failed to discriminate: when data from septic patients were analyzed, blood leukocyte counts, percents, or ratios did not distinguish survivors from non-survivors (Figures 1A–F). CRP data also overlapped across categories (Figures 1G,H).

**Figure 2:**
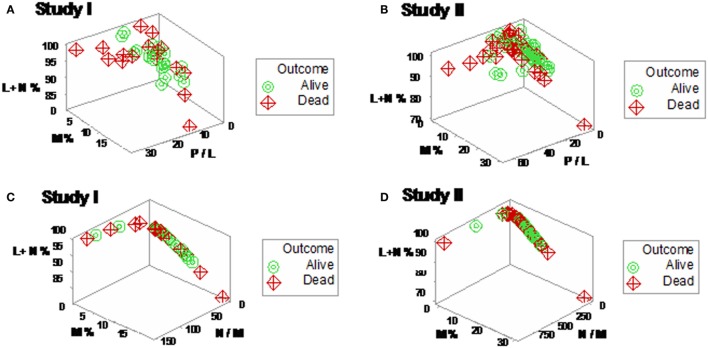
**Elimination of non-informative patterns**. The combinatorial and three-dimensional (3D) method was not always informative: many plots did not show distinct patterns **(A,B)**, even when a single (one data point-wide) line of observations was detected **(C,D)**.

When 3D plots were utilized, not all plots were informative (Figures 2A,B). Even plots that showed perpendicular data inflections did not predict, based on admission data, outcomes observed 30 days later (Figures 2A–D).

**Figure 3:**
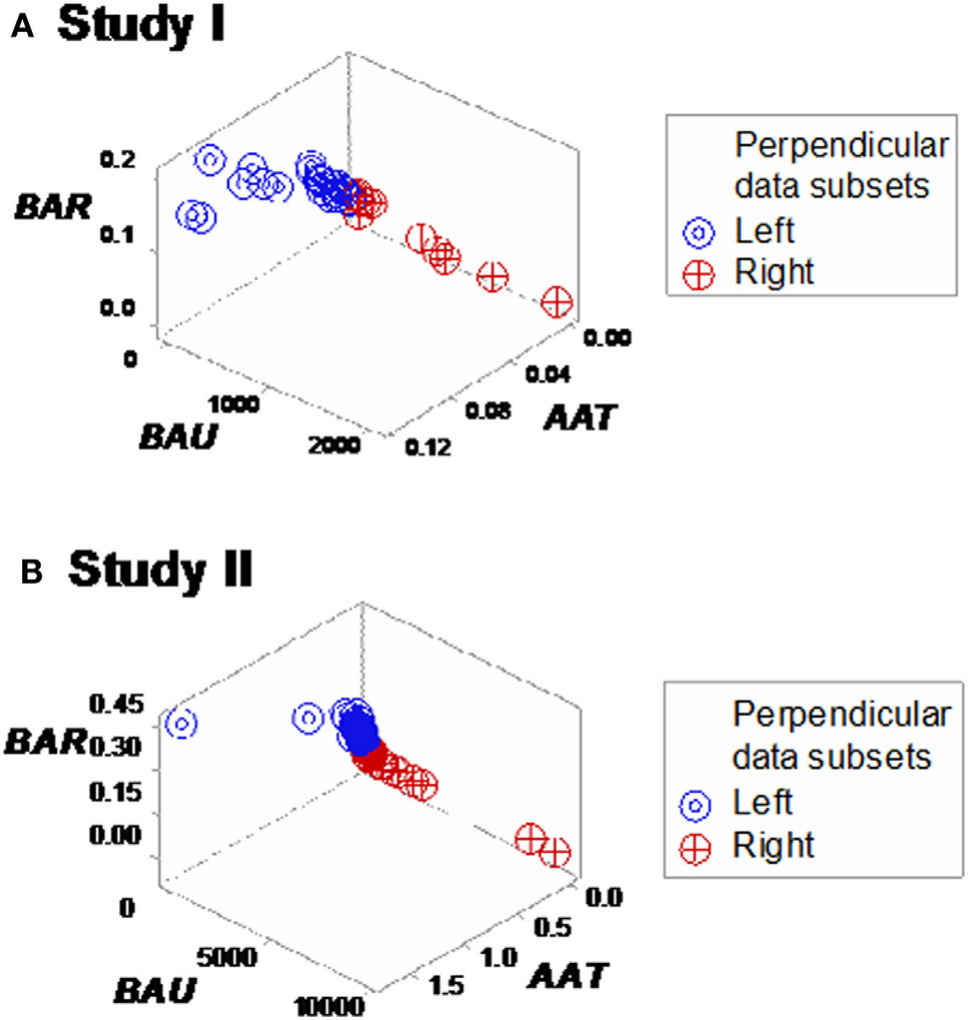
**Three-dimensional (combinatorial and partitioning-oriented) analysis of dimensionless indicators derived from leukocyte data (structure I)**. The large number of combinations the alternative method can generate increased the likelihood of finding some informative plots. Both study I **(A)** and II **(B)** displayed a single (one data point-wide) line of observations, which consisted of two segments perpendicular to one another. Such graphic data structure improves detection because data points can only occur along the line.

**Figure 4:**
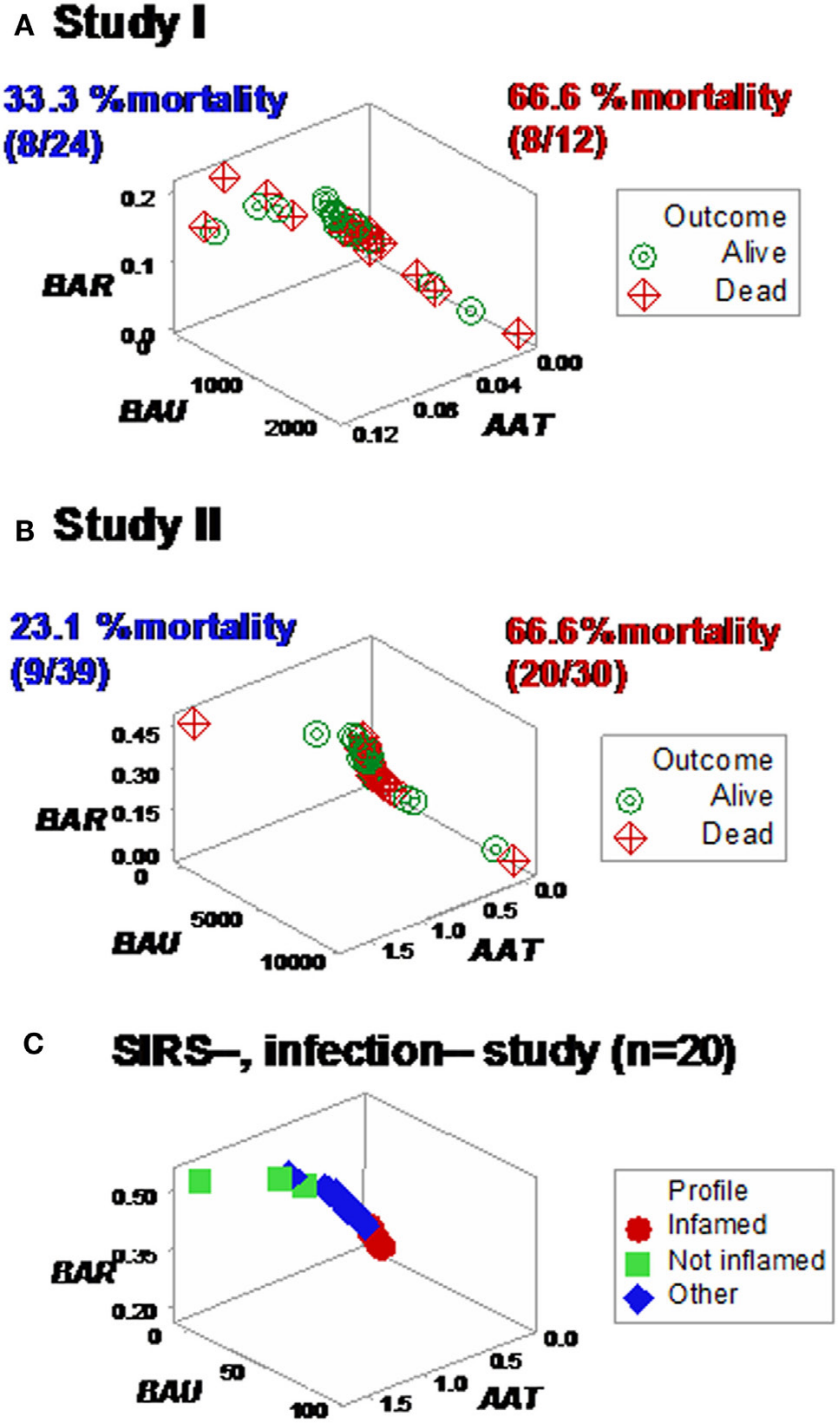
**Mortality rates of perpendicular data segments**. Both study I **(A)** and II **(B)** displayed mortality rates at least twice higher in the subset located on the right side of the plot than in the left subset (66.6% in both studies vs. 33.3 or 23.1%, in study I or II, respectively). Such differences approached or achieved statistical significance [*P* = 0.057 (study I) or *P* ≤ 0.01 (study II), Chi-square test]. When the same data structure was utilized to analyze 20 non-infected, SIRS-negative individuals, the high-mortality subset was not observed **(C)**, even though the scale of the critical axis (*BAU*) was 1000 times smaller than the scale used in **(A,B)**; i.e., the scale facilitated the detection of any pattern, if present.

Yet, some complex, 3D data structures showed a single (one data point-wide) line of observations, which revealed two data segments perpendicular to one another (Figures 3A,B). Therefore, at admission (before microbiological test results were completed), graphic patterns identified two data subsets. When, 2 days later, microbiological test results were available, neither bacterial species nor antibiotic susceptibility patterns explained the observed subsets (Figures S1 and S2 in Supplementary Material). In contrast, 30-day mortality was at least twice higher in the subset located at the right side of the plot (Figures 4A,B). While SIRS-negative, non-infected subjects differed in several aspects (Figure S3 in Supplementary Material), data from such individuals did not show the high-mortality pattern (Figure 4C).

**Figure 5:**
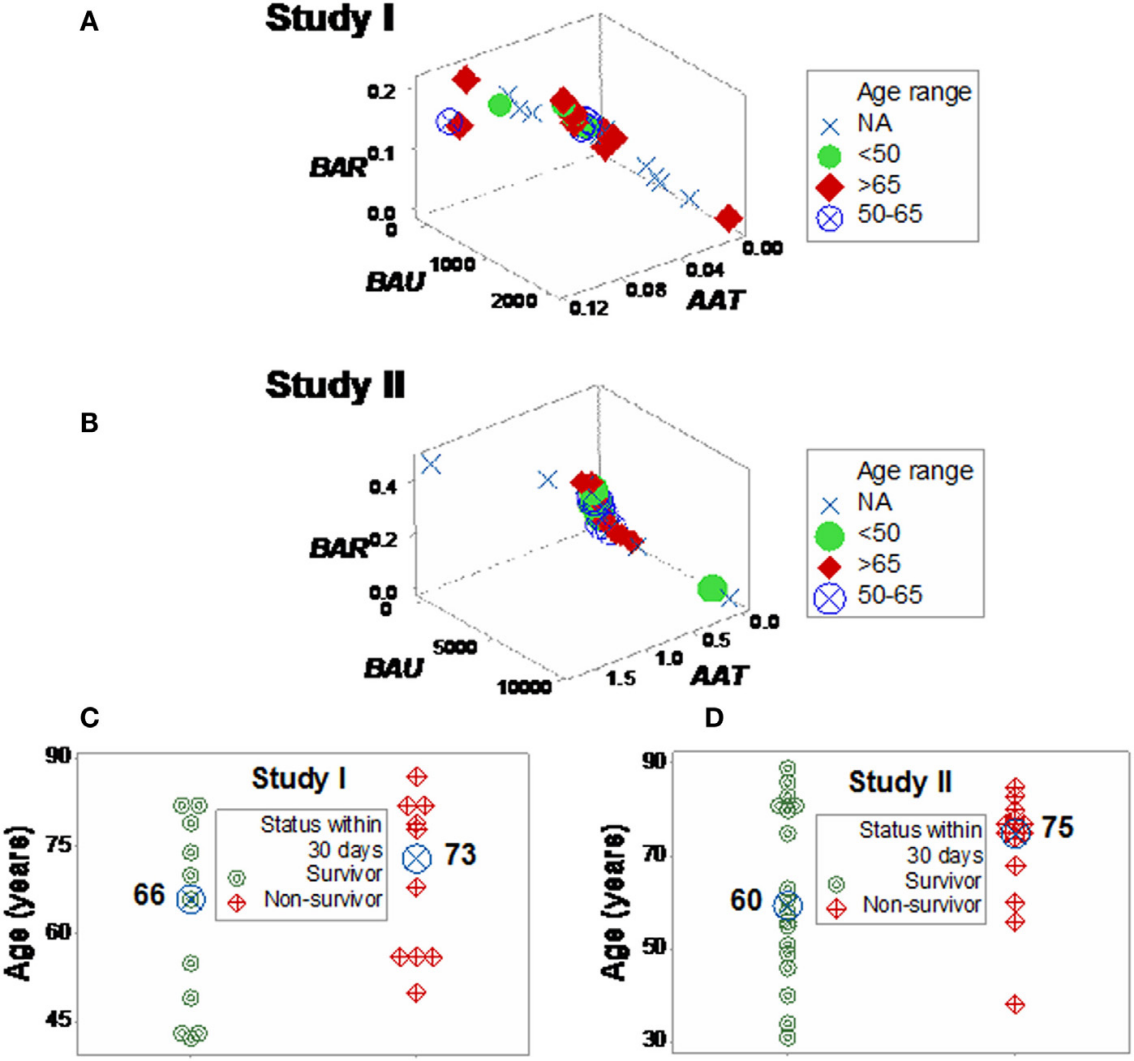
**Three-dimensional analysis of age data**. The age of SIRS+, infected individuals did not explain the mortality rates described in Figure 4. Both study I **(A)** and II **(B)** included >65-year-old individuals in the left (low-mortality) subset and <50-year-old individuals in the right (high-mortality) subset. While the median age [large, blue circles **(C,D)**] was 7–15 years higher in the right subset, any age-based cutoff would result in a large number of errors because the age intervals of survivors and non-survivors overlapped **(C,D)**.

Neither age nor gender explained mortality (Figures 5A,B; Figure S4 in Supplementary Material). While the median age was higher in the high-mortality subset, the distribution of age values overlapped between the survivor and non-survivor groups, preventing an age-based differentiation of the two subsets (Figures 5C,D).

**Figure 6:**
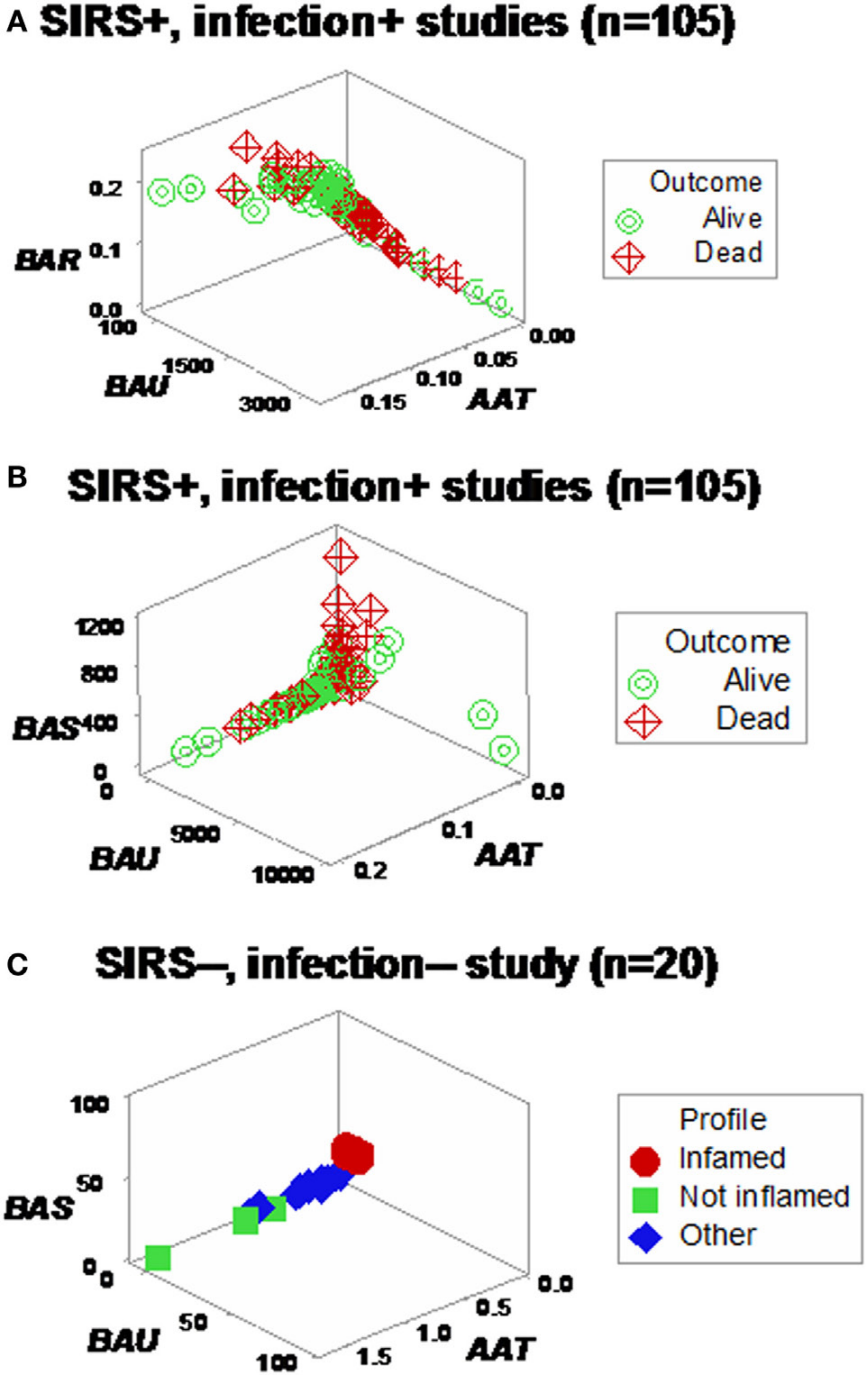
**Assessment of redundancy (structure II)**. When all 105 SIRS+, infection+ individuals were assessed, two (“left” and “right”) perpendicular subsets were observed, which differed in mortality rates: it was 26.1% in the left subset and 54.2% in the right subset (*P* < 0.004, Chi-square test, Table 1). For clarity, the *X* and *Y* axes are scaled down, and three data points are not plotted **(A)**. To prevent errors and improve the chances of extracting more information from the same data, an additional data structure was investigated, which also showed two data subsets orthogonal to one another **(B)**. In contrast, SIRS-negative, non-infected individuals did not present the high-mortality pattern, even though the scale of the *Y* axis (*BAU*) was 1000 times smaller than the scale used when SIRS+, infection+ individuals were tested **(C)**.

Different shapes –that also revealed a perpendicular data inflection –were seen when both SIRS+, infected groups were assessed with two partially different data structures (Figures 6A,B). Regardless of the data structure used, mortality was higher in the subset located at the right side of the plot [*P* < 0.001, Chi-square test (*n* = 105), Table 1]. The high-mortality subset was not observed when non-infected, SIRS-negative individuals were analyzed (Figure 6C).

**Table 1:** **Mortality rate per subset**.

Population	Outcome	Left subset	Right subset	Pearson Chi-square test
Both studies (*n* = 105)	Survivor	34	27	
	Non-survivor	12	32	
	Subtotal	46	59	
	Mortality (%)	26.1% (12/46)	54.2% (32/59)	*P* < 0.004
Study I (*n* = 36)	Survivor	16	4	
	Non-survivor	8	8	
	Subtotal	24	12	
	Mortality (%)	33.3% (8/24)	66.6% (8/12)	*P* = 0.057
Study II (*n* = 69)	Survivor	30	10	
	Non-survivor	9	20	
	Subtotal	39	30	
	Mortality (%)	23.1% (9/39)	66.6% (20/30)	*P* < 0.001

**Figure 7:**
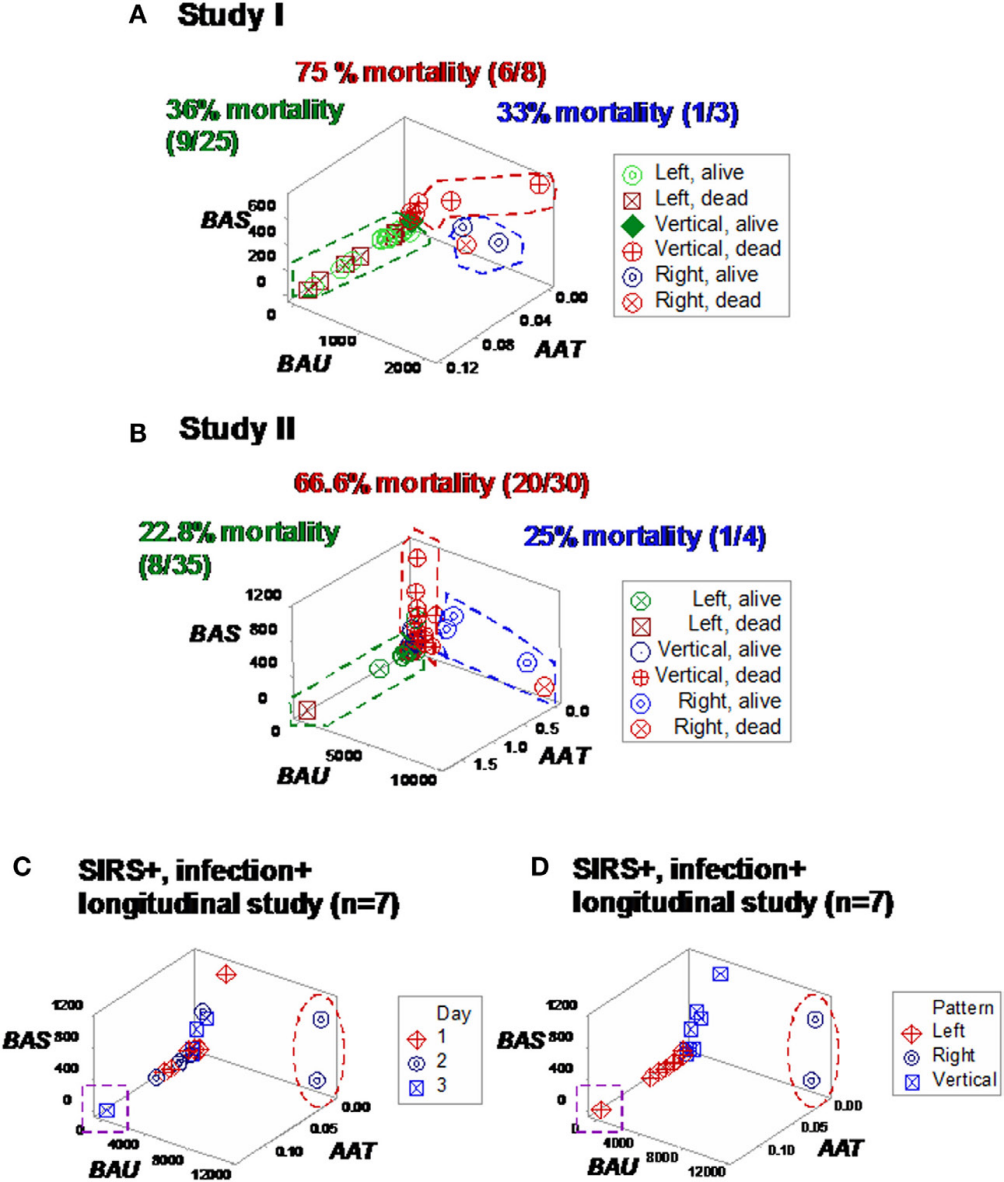
**Study-specific, subset-specific assessment of mortality**. When the second data structure was analyzed in each study, three spatial patterns were observed, both in study I **(A)** and II **(B)**, which showed differences in mortality. When an additional group of seven septic (SIRS+, infection+) individuals was tested over time, three patients were distinguished at day 2 or 3, who displayed (i) a “right” pattern [observable at day 2, two patients (red oval)] or (ii) a “left” pattern [detected at day 3, one patient (purple square) **(C)**]. When time was not considered, spatial patterns identified three subsets, classified as “left,” “vertical,” and “right” **(D)**.

**Figure 8:**
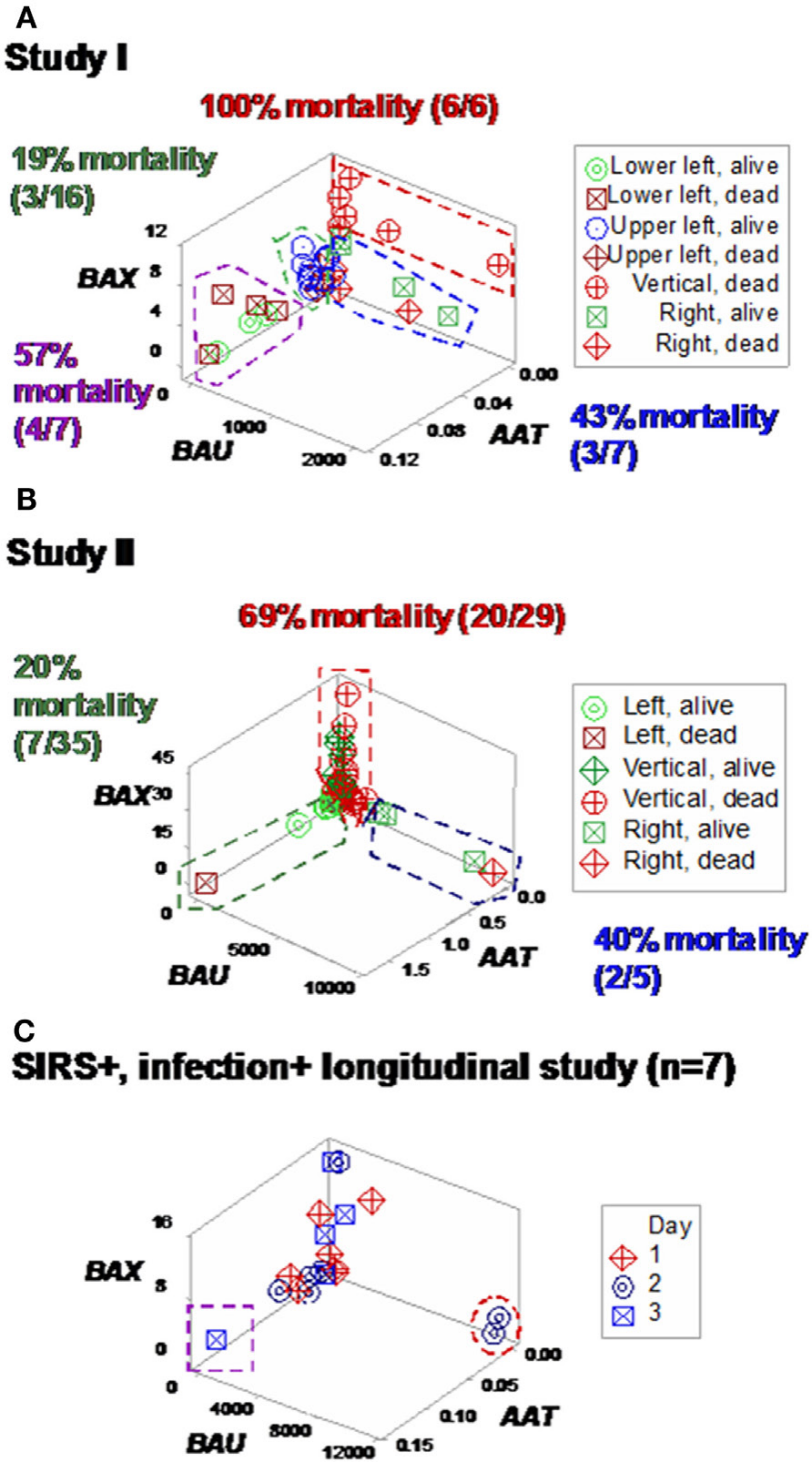
**Study-specific, sub-specific assessment of mortality (structure III)**. At least three profiles were distinguished when a third data structure was explored **(A,B)**. Mortality differed up to five times across subsets. Longitudinal data of septic patients displayed similar patterns, distinguishing at least three individuals [red oval, purple square **(C)**].

Some plots detected three subsets in each SIRS+/infection+ study, which differed up to three times in fatalities (Figures 7A,B). Longitudinal analysis of seven SIRS+/infected patients also showed perpendicular data subsets, which, within 24 h, helped differentiate patients (Figures 7C,D). Three data subsets were also displayed by a third set of indicators (Figures 8A–C).

**Figure 9:**
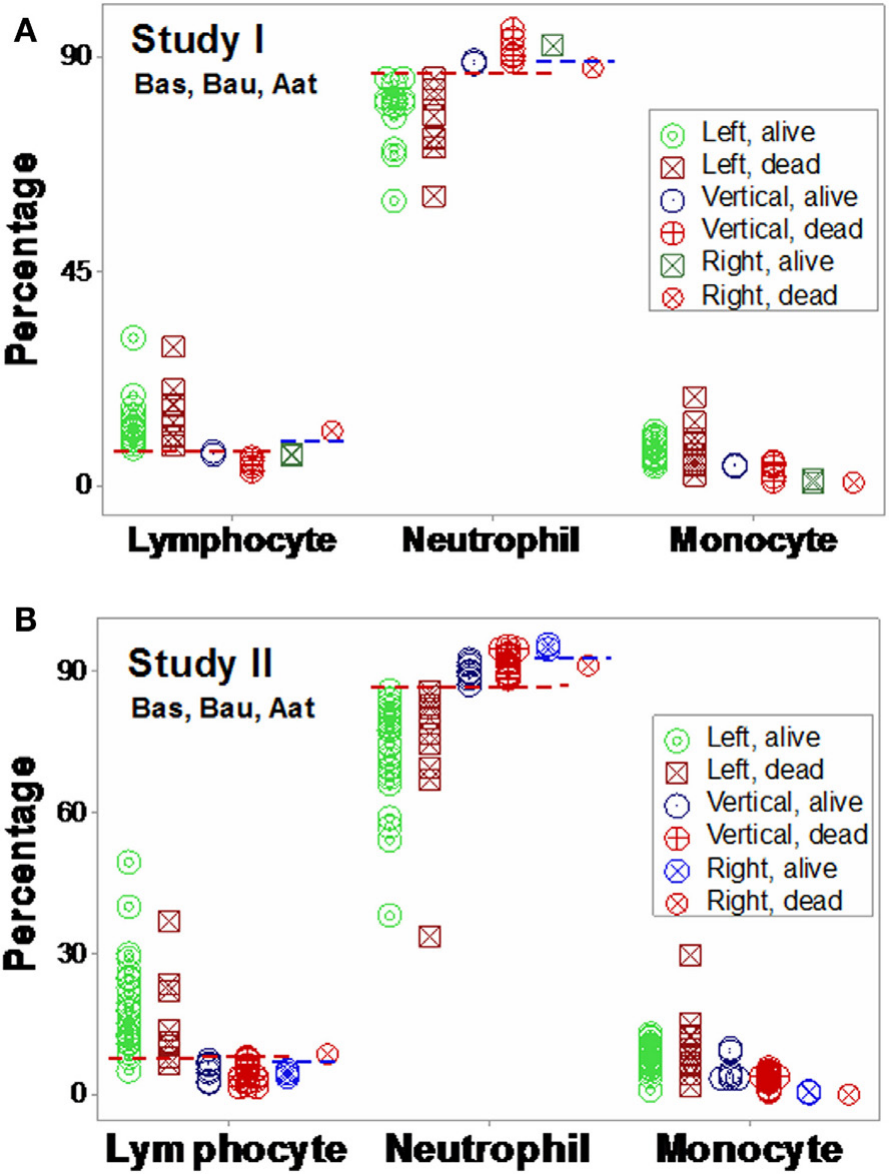
**Validation of subsets detected by structure II**. The immune profiles of subsets detected in Figures 7A,B were investigated. Both study I **(A)** and study I **(B)** showed non-randomly distributed leukocyte profiles, both within- and between-subsets. For instance, in both populations, *within-subset* differences were observed in the “right” subset, where the lymphocyte and neutrophil percentages did not overlap between survivors and non-survivors [blue horizontal lines **(A,B)**]. *Between-subset* differences were also observed, e.g., “left” subset survivors displayed higher L%, higher M%, and lower N% than survivors classified within the remaining subsets [red horizontal lines **(A,B)**]. Horizontal lines show some data subsets that did not overlap.

**Figure 10:**
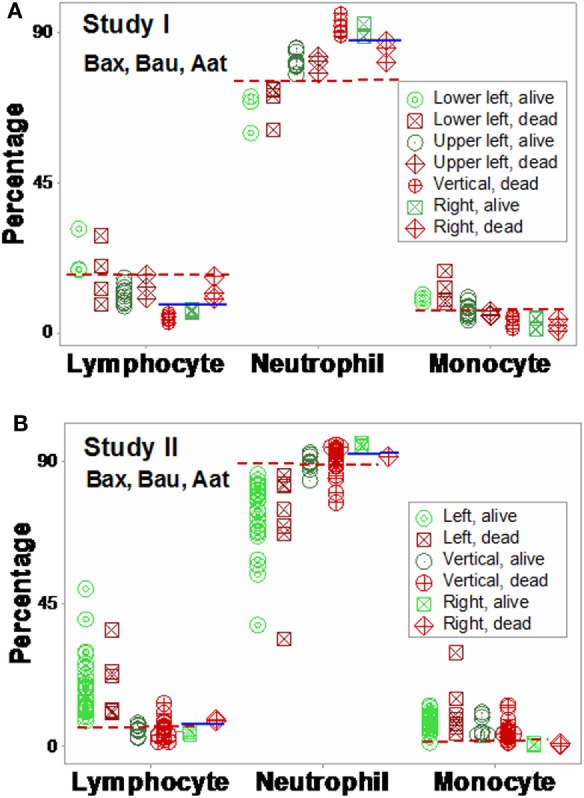
**Validation of subsets detected by structure III**. The immune profiles of subsets detected in Figures 8A,B were investigated. Discrimination was repeatable, even when a different data structure was utilized. While patterns differed slightly [study I (*n* = 36) showed four subsets, and one pattern (“vertical”) was only composed of one outcome (non-survivors) **(A)**], study II (*n* = 69) detected three subsets, with two outcomes per subset **(B)**. The “left” and “right” subsets of both studies reproduced the information observed in Figure 8: while the “left” subset did not show within-subset differences, the lymphocyte and neutrophil percentage intervals of the “right” subset did not overlap. The “vertical” subset of study II seemed to correspond to both the “vertical” and “lower left” subsets of study I. Horizontal lines show some data subsets that did not overlap.

Based on the three (“left,” “vertical,” and “right”) subsets identified in Figures 7A,B, subset-specific immune response profiles were analyzed (Figures 9A,B and 10A,B). *Within-subset* differences were observed in the “vertical” and “right” subsets. For instance, “right” survivors and non-survivors displayed non-overlapping lymphocyte and neutrophil distributions (Figures 9A,B). *Between-subset* differences were found in the “left” and “right” data groups, which displayed similar mortality rates (ranging between 22.8 and 36%, Figures 7A,B), but dissimilar (and non-overlapping) neutrophil and monocyte percentages (Figures 9A,B).

Mortality was not always predicted by numerical data. For instance, low lymphocyte percentages were found both among survivors (“right” subset) and among non-survivors (“vertical” subset, Figures 9A,B). Discrimination was not data structure-specific: similar findings were observed when a different data structure was investigated (Figures 10A,B).

**Figure 11:**
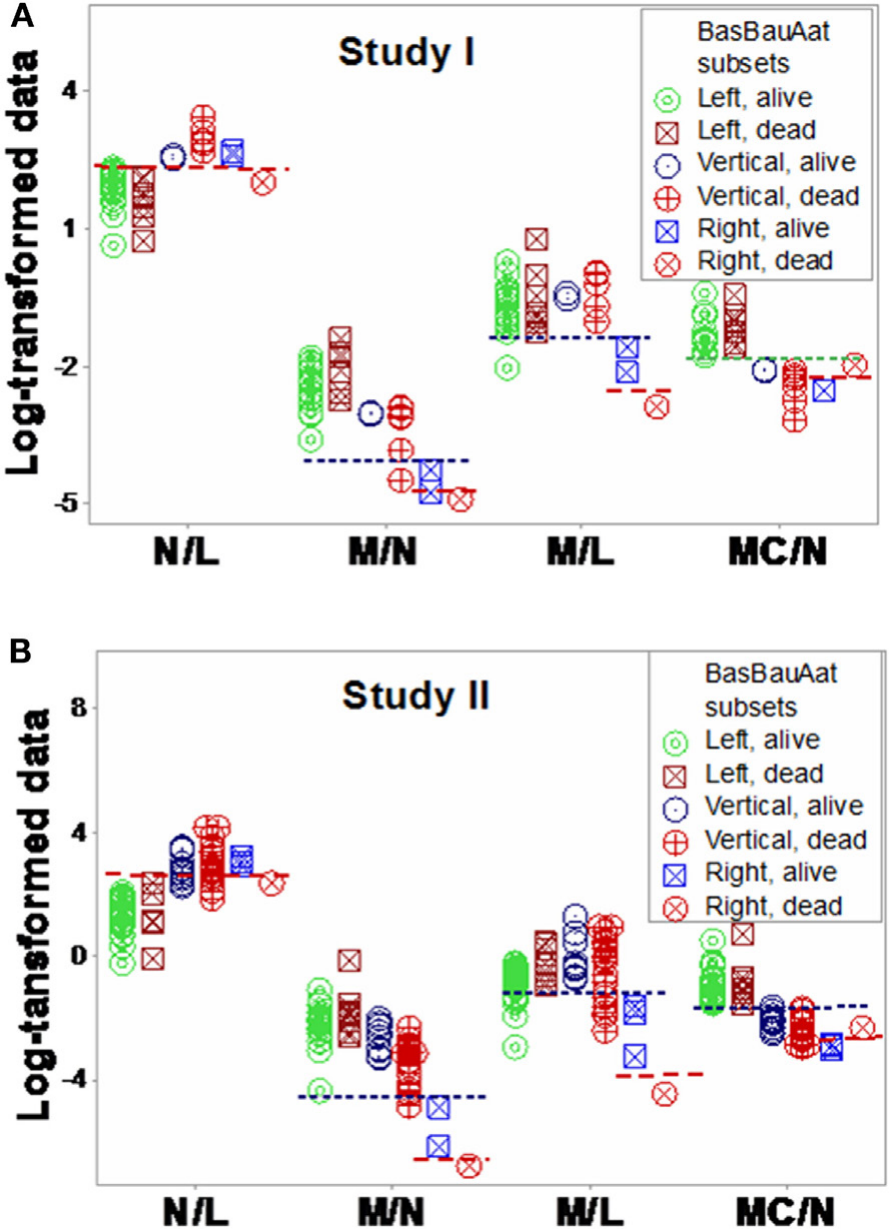
**Assessment of immune functions**. Based on the subsets detected in Figures 7A,B, interactions involving two or three cell types were investigated. Both study I **(A)** and study II **(B)** conveyed similar information. Both survivors and non-survivors of the “left” subset displayed the lowest neutrophil/lymphocyte (N/L) and highest mononuclear cell/neutrophil (MC/N) values. The “right” subset showed the lowest monocyte/neutrophil (M/N) and monocyte/lympohocyte (M/L) values, as well as within-subset differences were not observed in the remaining subsets: “right” non-survivors revealed lower N/L, M/N, and M/L and higher MC/N values than “right” survivors. The “vertical” non-survivors showed the highest N/L and the lowest MC/N values of all non-survivors. Together with the information shown in Figures 9A,B, these patterns support three hypotheses (i) the “right” subset experienced a monocyte-mediated immunosuppression; (ii) the “vertical” subset expressed excessive inflammation, together with low lymphocyte percentages; and (iii) mortality was not due, in the “left” subset, to any of the three disorders observed in the other subsets.

Additional *within*- and *between-subset* differences were noticed when ratios that included data from two or three cell types were evaluated. For instance, “vertical” non-survivors exhibited the highest neutrophil/lymphocyte (N/L) values among all subsets. In contrast, “right” non-survivors displayed lower N/L, lower monocyte/neutrophil (M/N), and lower monocyte/lymphocyte (M/L) values than “right” survivors (Figures 11A,B).

**Figure 12:**
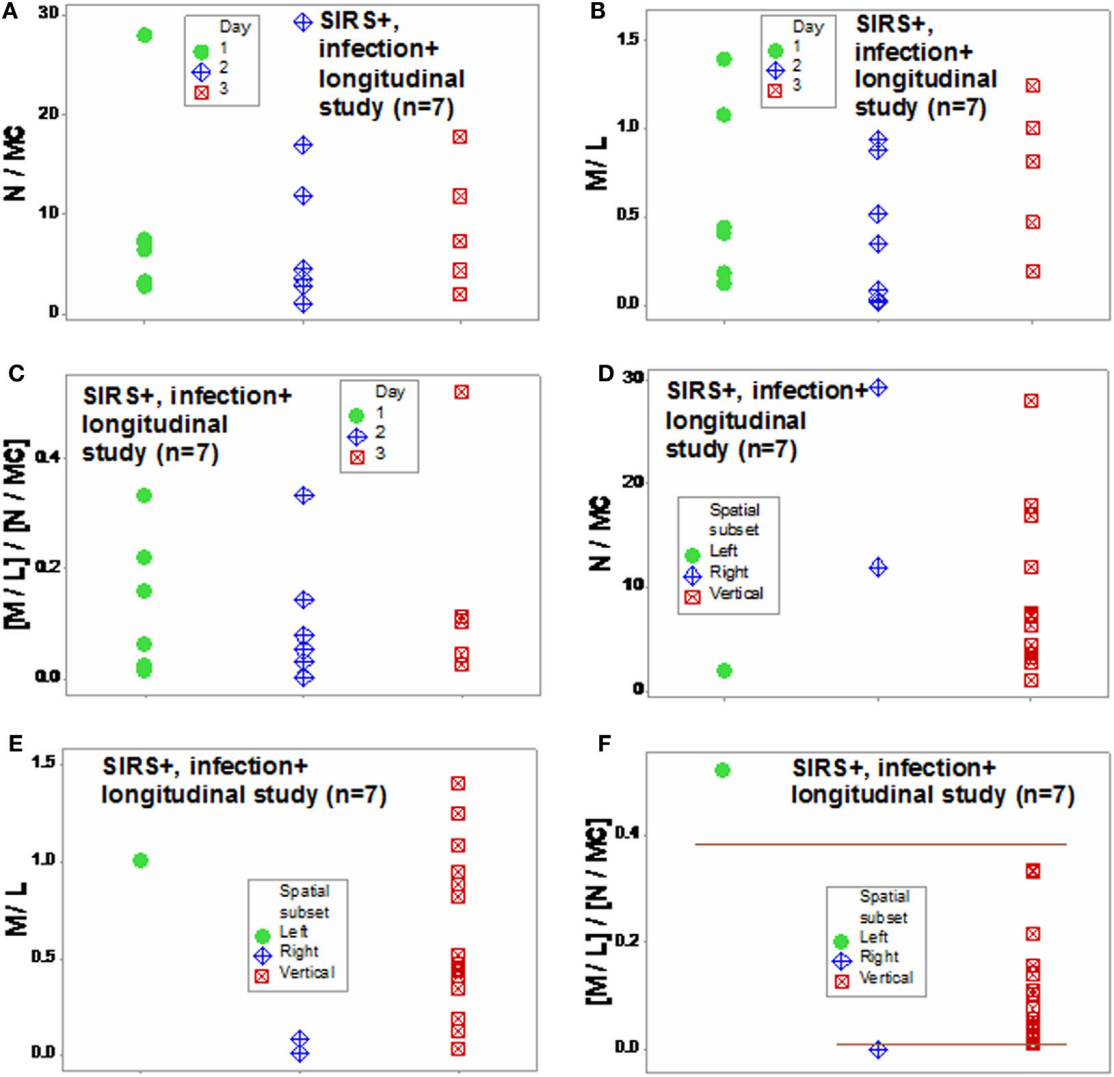
**Assessment of disease dynamics**. The combinatorial approach also measured disease dynamics, expressed as temporal interactions that included antibiotic–microbial–immunological relationships. Temporal information (days after admission) was not informative when either one or two levels of interactions were measured [N/MC, M/L, and (M/L)/(N/MC) **(A–C)**]. One-level interactions also failed to discriminate when the spatial patterns shown by Figure 7D were considered **(D,E)**. In contrast, non-overlapping data distributions were observed when both spatial profiles and two-level interactions were assessed, confirming the expectation that discrimination increases when two or more levels of complexity are investigated **(F)**. Horizontal lines denote non-overlapping data distributions **(F)**.

Similar patterns were observed in the longitudinal study of septic patients. While no discrimination was achieved when either information on days or indicators that captured one level of interactions [M/L, neutrophil/mononuclear cell (N/MC) ratios, Figures 12A–F] was assessed, the analysis of two-level interactions [(M/L)/(N/MC)] showed non-overlapping data distributions (Figure 12F). Therefore, the profile shown by one “left” and two “right” patients –identified in Figures 7C and 8C –was at least partially characterized by either (i) a rather late inflammatory profile [low levels of neutrophils/mononuclear cells (low N/MC values), and/or high M/L values, in the “left” patient] or (ii) the opposite profile, revealed by two “right” patients.

While the data (reported in Tables S1–S4 in Supplementary Material) were not informative prior to data partitioning, after data partitioning, many *within*- and *between*-subset comparisons achieved statistical significance (Tables S4–S8 in Supplementary Material). Therefore, the hypothesis that hidden information may be embedded in blood cell data was supported.

## Discussion and Conclusion

Infectious disease-related research, including associated syndromes (such as sepsis), may benefit from methods that assess immunological complexity (41). Accordingly, a method meant to estimate complexity was developed and tested. Because it did not assume that the whole can be reduced to or predicted from any part, the combinatorial approach was a *non-reductionist* method. In contrast, the analysis shown in Figures 1A–H reflected a *reductionist* approach –it assumed that the analysis of isolated parts could predict or separate outcomes (6, 19, 20). While the reductionist, non-partitioning approach failed to discriminate, the non-reductionist, combinatorial method identified and partitioned data subsets that differed in mortality.

While previous blood cell count-based studies have failed to predict mortality (29, 42), earlier studies had assumed that lymphocytes, neutrophils, or monocytes act independently and, accordingly, such cell types had been analyzed separately. In contrast, the combinatorial method assessed all the observations of all cell types, together.

Hidden data interactions were demonstrated and septic patients were grouped according to their immune profiles. In addition, several problematic properties of infectious disease data were prevented, including those associated with *ambiguity* and *compositions*. For example, discrimination was achieved without depending on the white blood cell count (one of the four SIRS criteria), which may be affected by the features of compositional data (15, 36).

While counts, percentages, and simple ratios were not informative *per se*, when integrated into data structures that captured *several levels of complexity*, new or more information was retrieved. Because some complex data structures revealed a single (one data point-wide) line of observation, personalized applications were fostered: when any pair of observations –collected over time, from the same individual –are analyzed on a single line, they will exhibit a movement (temporal data directionality), indicating whether the later observation approaches the disease-negative or -positive pole of the data. Such structure prevents data ambiguity because a single line of data point eliminates noise (data variability) from all dimensions, except along the line, while the information reported along the line facilitates (i) monitoring of disease progression (dynamics) and (ii) evaluation of therapies (1, 43).

By detecting data subsets according to graphic patterns, inferences were made without assumptions, and dichotomization was avoided (24). In agreement with reports that have indicated no single biomarker is likely to be diagnostic in personalized medicine (44), analyses that included a single biomarker (CRP) were not informative (Figures 1G,H).

Inferences were based on visualizations that reflected both interactions and dynamics. Here, “interactions” were operationally defined as patterns generated when three variables intersect in 3D space: when one variable increases or decreases in a larger magnitude than the remaining variables, a distinct pattern emerges, e.g., a data inflection, which can be used to separate data subsets (45). Such inflections or bifurcations can reveal a common feature of infections: *dynamics*. Because homeostatic (feedback and feedforward) processes also occur in infections, they can be used to provide new diagnostic and prognostic information (1, 46).

While some feedback phases may change rapidly and reveal non-linearity (46), the subsets they generate may be perpendicular to one another, as documented here. Thus, infection-related feedback processes may generate a biological equivalent of what, in mathematics, is created by a log-transformation: data, inherently non-linear, can be treated as linear and, consequently, after data subsets perpendicular to one another are observed, statistical analyses can be conducted. When similar data subsets are observed in different populations, the hypothesis of a random event is not defensible –instead, a well-conserved pattern is likely. In such a case, the “transformation” of non-linear immunological data into perpendicular data subsets is not the result of an equation but produced by a well-conserved biological process.

The combinatorial approach also provided *explanatory* information, of immunological nature, which may support diagnostics and therapy selection (47). Clinicians working in sepsis need new diagnostics: current diagnostic criteria have shown very poor (less than 50%) sensitivity values (48, 49). Clinicians could benefit from *earlier* evaluations of diagnostic and therapeutic decisions. As shown here, the dynamics of seven septic patients differed over 3 days: two patients revealed a “right” pattern as early as day 2 and (at least) one patient displayed the “left” pattern by day 3 (Figures 7C and 8C). While other factors, not explored (such as the role of empirical antibiotic treatments), prevent to elucidate whether the two “right” patients experienced a better or worse disease progression and/or responsiveness to treatment than the remaining patients, Figure 12F demonstrates that the combinatorial approach can monitor disease dynamics *earlier* (1 day before *in vitro* tests are completed), based on *in vivo*, temporal data that assess not only immunological but also antibiotic–microbial relationships.

The future of therapy, in sepsis, has been indicated to depend on the identification of immune phases or profiles (50–52). Sepsis seems to be a systemic response to infections, which is associated with organ dysfunction (53). Supporting earlier reports, findings revealed dissimilar immune profiles among subsets that also differed in mortality rates (54).

“*Vertical*” *non-survivors* displayed an excessive inflammatory response as well as low lymphocyte percentages. When the 3D patterns exhibited by Figures 7A,B were considered, individuals classified within the “vertical” subset displayed higher N% and lower L% than those of the “left” subset (Figures 9A,B). “Vertical” non-survivors showed the highest neutrophil/lymphocyte (N/L) values of all subsets and also higher monocyte/lymphocyte (M/L) values than those of the “right” subset (Figures 11A,B), that is, the “vertical” non-survivor profile was consistent with enhanced lymphocyte apoptosis and delayed monocyte apoptosis; i.e., an immunopathology that may result in protracted inflammation and immunosuppression (55–61).

“*Right*” *non-survivors* –unlike the “vertical” ones –exhibited a profile that did not support the hypothesis of mortality induced by low lymphocyte values and excessive inflammation. Instead, “right” non-survivors displayed higher L% than “right” survivors (Figures 9A,B and 10A,B). “Right” non-survivors did not seem to prevent neutrophil recruitment: they showed higher N% than “left” non-survivors (Figures 9A,B and 10A,B). Because “right” non-survivors expressed lower (if not the lowest) M% than observed in all other subsets, a monocyte-related deficiency could be associated, in this subset, with mortality. Homeostatic failures associated with macrophage deficiencies include (i) the limited expression of macrophage-antigen 1 (Mac-1) and (ii) decreased expression of CD11b in dendritic cells (55, 62, 63). Such disruptions can alter trans-membrane permeability and complement fixation functions, without preventing neutrophil release. Furthermore, the “right” subset documented that low lymphocyte values do not characterize all septic cases (51): “right” survivors displayed significantly lower lymphocyte percentages than “left” survivors (Figures 9A,B and Table S7 in Supplementary Material).

“*Left*” *survivors and non-survivors* displayed the highest mononuclear cell/neutrophil (MC/N) values observed in this report, differing markedly from the remaining subsets (Figures 11A,B). Unlike other subsets (which revealed several *within-subset* differences), “left” survivors and non-survivors did not show obvious differences.

The “left” pattern supported three inferences (i) the theory that sepsis may be composed of four stages (sepsis, severe sepsis, septic shock, and refractory septic shock) may be clinically factual, but not scientifically informative; (ii) the proposition that the type of infection determines the outcome of sepsis may not always occur; and (iii) mortality, in the “left” subset, was not explained by the interactions investigated here. Because high MC/N values are typical of late or recurrent inflammations –and they were observed at admission, in the “left” subset –four-stage, clinical-based classifications may miss a late or recurrent process (62).

The “left” profile did not support the hypothesis that the type of infection determines the outcome of sepsis (43). Instead, the opposite view may occur: the immune profile (considered in its broadest meaning, i.e., including homeostatic perspectives) may influence the speed and outcome of antimicrobial responses. In support of the immune-mediated disease hypothesis, *Candida* sp. and *Pseudomonas* sp. (opportunistic microbes) infected two individuals classified within the “left” profile (Tables S1 and S2 in Supplementary Material). Because sepsis can occur without apoptosis or necrosis, disorders not typically viewed as inflammatory (e.g., mitochondrial dysfunctions that affect intracellular junctions) cannot be ruled out in the “left” profile, even in the absence of cellular deficits or altered responses (64).

Together, findings supported the view that at least two types of immunosuppression may be found in sepsis: coexisting and not coexisting with excessive inflammation. Two types of immunosuppression have been previously reported in sepsis (65).

Available at admission, this information could support therapy selection. While cytokines that boost the immune response (e.g., granulocyte–macrophage colony-stimulating factor and interleukin-3) may be of interest when the “right subset” profile is observed (52, 66), they may be inadequate in “vertical” immunosuppressions, which coexist with inflammation.

In sum, methods that assess immune complexity appeared to distinguish some sepsis-related sub-syndromes, in real time. Because diagnostic expediency was prioritized, the variables utilized did not cover all biological scales, e.g., cytokines were not investigated. Future studies could assess subcellular variables, as recently described (37).

Offering an alternative to the white blood cell count (a metric prone to ambiguity), complexity-oriented analyses detected and differentiated immunosuppressions. Such analyses can identify patients that differ in mortality risks and help conduct repeated evaluations of diagnosis and therapies. Because immunosuppression matters in infections and also in cancer and transplantation (67, 68), combinatorial analyses may have broad applications.

Two major messages result from this study. The first message refers to Oslerian medicine, that is, to define disease based on clinicopathological correlations derived from organs that express most signs and symptoms –even when disease does not start in such organs. The Oslerian paradigm confuses consequences with causes, resulting in late assessments that ignore pathogenesis (69). When this paradigm is applied with problematic practices –such as numerical cutoffs– and/or assumes that a single biomarker, alone, may capture complex and dynamic interactions, information loss and errors may follow.

The second message is that even leukocyte counts, percents, or simple ratios can be informative when composite metrics are used to explore complexity. While clinical descriptions may not be adequate to monitor disease progression and/or select therapies, immunomicrobial complexity may provide explanatory information, earlier.

## Author Contributions

The authors AR and GT conceived the study. SC, AI, ET-G, NC, CS, and TB contributed reagents/materials/data. AR, AH, ALH, and RP analyzed the data. AR, RD, and GT wrote the paper.

## Conflict of Interest Statement

While none of the authors received, at any time, any payment or services from a third party for any aspect of the submitted work, they wish to declare that they used a proprietary algorithm subject to a pending patent.
