# Supplementary material for: Detecting the Hidden Properties of Immunological Data and Predicting the Mortality Risks of Infectious Syndromes
Source: Front Immunol. 2016 Jun 10;7:217. doi: 10.3389/fimmu.2016.00217 (PMC4901050; doi:10.3389/fimmu.2016.00217)
Supplement: Supplementary file 1 [file Data_Sheet_1.doc]

**SUPPLEMENTARY MATERIAL**

**Table S1. SIRS+, infection+, study I (n=36)**

| Case  ID | Outc. | Sex | Age | WBC | CRP | L% | N% | M% | MS | AB res | Spatial subset* |
| --- | --- | --- | --- | --- | --- | --- | --- | --- | --- | --- | --- |
| 65 | alive | F | 42 | 6.2 | * | 13.6 | 81.0 | 5.3 | 3 | 1 | Left, alive |
| 196 | alive | M | 66 | 7.6 | 3.6 | 7.9 | 81.4 | 10.6 | 3 | 1 | Left, alive |
| 450 | alive | M | * | 12.1 | * | 15.0 | 80.2 | 4.7 | 9 | 1 | Left, alive |
| 4555 | alive | M | * | 7.1 | 123.0 | 31.0 | 59.8 | 9.0 | 3 | 1 | Left, alive |
| 496 | alive | F | * | 11.9 | * | 8.9 | 85.3 | 5.6 | 9 | 1 | Left, alive |
| 557 | alive | F | * | 8.7 | * | 11.7 | 84.2 | 4.0 | 3 | 1 | Left, alive |
| 568 | alive | M | 74 | 6.7 | 112.0 | 10.7 | 81.0 | 8.1 | 3 | 1 | Left, alive |
| 712 | alive | M | 55 | 13.0 | 85.9 | 9.2 | 84.3 | 6.3 | 3 | 1 | Left, alive |
| 735 | alive | M | 43 | 13.5 | * | 9.6 | 80.5 | 9.8 | 3 | 1 | Left, alive |
| 775 | alive | M | 82 | 8.5 | 40.9 | 12.6 | 79.7 | 7.5 | 3 | 1 | Left, alive |
| 781 | alive | M | 70 | 13.8 | 31.6 | 16.4 | 77.4 | 6.0 | 3 | 1 | Left, alive |
| 799 | alive | M | * | 6.6 | 90.8 | 18.9 | 70.7 | 10.2 | 2 | 1 | Left, alive |
| 828 | alive | F | 49 | 7.1 | * | 19.0 | 69.4 | 11.5 | 3 | 1 | Left, alive |
| 842 | alive | M | 82 | 11.7 | 20.4 | 11.8 | 80.1 | 8.0 | 10 | 0 | Left, alive |
| 945 | alive | M | * | 9.9 | * | 8.7 | 85.3 | 5.8 | 4 | 1 | Left, alive |
| 980 | alive | M | 43 | 13.6 | 136.0 | 12.2 | 80.1 | 7.5 | 3 | 1 | Left, alive |
| 406 | dead | F | * | 13.9 | * | 10.3 | 85.3 | 4.2 | 11 | 1 | Left, dead |
| 81 | dead | F | 82 | 18.4 | 16.3 | 16.7 | 81.0 | 2.2 | 3 | 1 | Left, dead |
| 273 | dead | M | 56 | 8.4 | 115.0 | 13.4 | 81.2 | 5.2 | 3 | 1 | Left, dead |
| 334 | dead | F | * | 8.4 | * | 13.3 | 73.1 | 13.4 | 5 | 1 | Left, dead |
| 434 | dead | M | 56 | 13.1 | * | 17.2 | 77.7 | 4.9 | 3 | 1 | Left, dead |
| 552 | dead | F | 82 | 10.7 | 24.8 | 8.5 | 72.8 | 18.6 | 3 | 1 | Left, dead |
| 911 | dead | M | 56 | 9.9 | * | 10.4 | 82.9 | 6.5 | 3 | 1 | Left, dead |
| 922 | dead | M | 50 | 3.6 | 12.6 | 29.0 | 60.8 | 10.1 | 3 | 1 | Left, dead |
| 953 | dead | F | * | 10.3 | 57.6 | 20.0 | 71.0 | 8.8 | 1 | 0 | Left, dead |
| 554 | alive | F | * | 14.9 | * | 6.5 | 89.0 | 4.4 | 5 | 1 | Vertical, alive |
| 647 | alive | M | * | 6.5 | * | 7.0 | 88.7 | 4.2 | 5 | 1 | Vertical, alive |
| 187 | dead | M | * | 12.9 | * | 4.0 | 93.8 | 2.0 | 7 | 1 | Vertical, dead |
| 202 | dead | M | 78 | 7.7 | 91.2 | 4.0 | 91.8 | 4.1 | 3 | 1 | Vertical, dead |
| 254 | dead | M | 79 | 9.8 | 31.2 | 3.0 | 95.8 | 1.1 | 3 | 1 | Vertical, dead |
| 454 | dead | M | 87 | 15.3 | * | 5.2 | 90.5 | 4.2 | 3 | 1 | Vertical, dead |
| 801 | dead | M | 68 | 6.8 | * | 5.9 | 89.1 | 4.8 | 3 | 1 | Vertical, dead |
| 997 | dead | F | * | 10.9 | 72.1 | 4.8 | 90.0 | 5.1 | 6 | 1 | Vertical, dead |
| 296 | alive | M | * | 4.5 | 152.0 | 6.7 | 92.4 | 0.8 | 12 | 1 | Right, alive |
| 927 | alive | F | * | 17.3 | * | 6.2 | 92.4 | 1.3 | 9 | 1 | Right, alive |
| 238 | dead | F | * | 15.7 | * | 11.7 | 87.6 | 0.6 | 6 | 1 | Right, dead |

- Spatial subsets were classified according to the patterns described in Figure 7a.
- For instructions on how to reproduce analyses, see the footnote of Table S2

**Table S2. SIRS+, infection+, study II (n=69)**

| Case  ID | Outc | Sex | Age | WBC | CRP | L% | N% | M% | MS | AB res | Spatial subset* |
| --- | --- | --- | --- | --- | --- | --- | --- | --- | --- | --- | --- |
| 5 | alive | F | * | 17.5 | 219 | 2.9 | 92.5 | 4.6 | 3 | 1 | Vertical, alive |
| 25 | alive | F | 63 | 4.8 | 44 | 19.7 | 79.3 | 1.0 | 13 | 1 | Left, alive |
| 48 | dead | M | 75 | 24.0 | 24 | 3.7 | 95.3 | 1.1 | 9 | 0 | Vertical, dead |
| 92 | alive | M | * | 24.6 | * | 5.2 | 94.6 | 0.2 | 9 | 1 | Right, alive |
| 92 | alive | * | * | 7.0 | * | 3.7 | 95.6 | 0.7 | 19 | 1 | Right, alive |
| 139 | dead | M | 60 | 18.4 | 198 | 2.0 | 93.7 | 4.3 | 6 | 1 | Vertical, dead |
| 148 | alive | F | 56 | 14.2 | * | 11.1 | 79.9 | 8.9 | 3 | 1 | Left, alive |
| 193 | dead | M | * | 33.4 | 195 | 4.9 | 92.5 | 2.6 | 3 | 1 | Vertical, dead |
| 202 | dead | F | 85 | 18.8 | 258 | 2.6 | 93.5 | 3.9 | 6 | 1 | Vertical, dead |
| 211 | dead | M | * | 20.6 | * | 3.7 | 93.2 | 3.1 | 3 | 1 | Vertical, dead |
| 215 | dead | M | 38 | 9.6 | * | 10.3 | 85.5 | 4.2 | 4 | 1 | Left, dead |
| 216 | alive | F | * | 8.8 | 186 | 8.9 | 84.1 | 6.9 | 3 | 1 | Left, alive |
| 225 | dead | F | 68 | 9.3 | 137 | 7.8 | 91.5 | 0.7 | 11 | 1 | Vertical, dead |
| 241 | alive | M | * | 5.9 | * | 40.0 | 54.3 | 5.7 | 3 | 1 | Left, alive |
| 249 | alive | F | 81 | 12.4 | * | 12.2 | 80.5 | 7.3 | 6 | 1 | Left, alive |
| 266 | dead | M | 75 | 6.9 | * | 13.4 | 84.6 | 1.9 | 6 | 1 | Left, dead |
| 275 | alive | M | * | 12.3 | * | 15.1 | 81.1 | 3.7 | 9 | 1 | Left, alive |
| 291 | alive | F | 81 | 12.4 | 100 | 11.8 | 80.5 | 7.7 | 1 | 1 | Left, alive |
| 302 | dead | M | 74 | 5.9 | 143 | 7.2 | 91.7 | 1.1 | 9 | 1 | Vertical, dead |
| 321 | alive | M | 46 | 9.6 | * | 25.9 | 67.4 | 6.6 | 15 | 1 | Left, alive |
| 327 | alive | M | 51 | 11.1 | 238 | 8.0 | 85.8 | 6.2 | 5 | 1 | Left, alive |
| 329 | alive | M | 61 | 11.3 | 288 | 2.6 | 88.3 | 9.1 | 5 | 1 | Vertical, alive |
| 359 | dead | F | * | 20.6 | 205 | 7.6 | 88.3 | 4.1 | 3 | 1 | Vertical, dead |
| 412 | dead | F | 77 | 22.7 | 117 | 3.2 | 91.6 | 5.2 | 20 | 1 | Vertical, dead |
| 417 | alive | F | 83 | 11.2 | 61 | 11.5 | 82.0 | 6.5 | 9 | 1 | Left, alive |
| 418 | alive | F | * | 6.4 | 31 | 10.7 | 82.6 | 6.7 | 3 | 1 | Left, alive |
| 426 | alive | F | 86 | 10.2 | * | 4.5 | 94.8 | 0.7 | 23 | 1 | Right, alive |
| 429 | alive | M | 55 | 4.2 | * | 15.6 | 74.2 | 10.1 | 11 | 1 | Left, alive |
| 441 | dead | F | 56 | 18.0 | 83 | 6.2 | 92.5 | 1.2 | 21 | * | Vertical, dead |
| 480 | dead | M | * | 18.3 | 15 | 6.3 | 85.5 | 8.2 | 3 | 1 | Left, dead |
| 497 | alive | F | * | 5.0 | * | 24.5 | 66.2 | 9.3 | 3 | 1 | Left, alive |
| 523 | alive | M | * | 12.4 | 200 | 12.8 | 77.4 | 9.7 | 3 | 0 | Left, alive |
| 536 | dead | F | * | 12.0 | * | 5.5 | 93.0 | 1.4 | 3 | 1 | Vertical, dead |
| 539 | alive | M | * | 17.0 | 57 | 4.7 | 91.9 | 3.4 | 3 | 1 | Vertical, alive |
| 558 | alive | F | * | 8.0 | 64 | 25.0 | 69.5 | 5.4 | 3 | 1 | Left, alive |
| 567 | alive | F | 58 | 9.6 | 5 | 22.5 | 69.3 | 8.1 | 3 | 1 | Left, alive |
| 591 | dead | F | * | 9.0 | * | 22.1 | 69.4 | 8.5 | 3 | 1 | Left, dead |
| 604 | alive | M | * | 8.5 | 151 | 5.1 | 84.1 | 10.8 | 3 | 1 | Left, alive |
| 616 | alive | M | * | 5.7 | 110 | 21.5 | 68.0 | 10.6 | 3 | 1 | Left, alive |
| 617 | dead | M | * | 7.6 | 48 | 36.7 | 33.8 | 29.5 | 3 | 1 | Left, dead |

**Table S2. SIRS+, infection+, group II (n=69, CONTINUED)**

| Case  ID | Outc. | Sex | Age | WBC | CRP | L% | N% | M% | MS | AB res | Spatial  subset* |
| --- | --- | --- | --- | --- | --- | --- | --- | --- | --- | --- | --- |
| 636 | alive | F | * | 12.3 | 52 | 6.6 | 89.9 | 3.4 | 10 | * | Vertical, alive |
| 642 | alive | M | 31 | 21.0 | * | 12.4 | 79.1 | 8.5 | 16 | 1 | Left, alive |
| 678 | alive | M | 89 | 19.6 | * | 2.8 | 87.3 | 9.9 | 17 | * | Vertical, alive |
| 686 | alive | F | 49 | 9.8 | * | 7.3 | 89.2 | 3.5 | 18 | 1 | Vertical, alive |
| 689 | alive | F | * | 10.1 | 59 | 13.9 | 79.8 | 6.3 | 3 | 1 | Left, alive |
| 734 | alive | M | 75 | 10.1 | * | 12.5 | 78.1 | 9.5 | 9 | 1 | Left, alive |
| 747 | dead | F | * | 9.5 | 160 | 23.3 | 67.1 | 9.7 | 3 | 1 | Left, dead |
| 783 | dead | M | * | 12.6 | * | 1.5 | 94.6 | 3.9 | 5 | 1 | Vertical, dead |
| 787 | alive | M | * | 6.3 | 172 | 17.8 | 77.3 | 4.9 | 3 | 1 | Left, alive |
| 802 | dead | M | * | 12.6 | * | 1.5 | 94.6 | 3.9 | 3 | 1 | Vertical, dead |
| 804 | dead | M | * | 12.1 | * | 5.6 | 89.8 | 4.6 | 3 | 1 | Vertical, dead |
| 810 | dead | F | 83 | 5.9 | * | 3.2 | 93.8 | 3.0 | 17 | * | Vertical, dead |
| 824 | dead | M | 78 | 17.3 | * | 5.6 | 88.7 | 5.7 | 22 | 0 | Vertical, dead |
| 833 | alive | M | * | 6.0 | 46 | 18.7 | 71.5 | 9.8 | 3 | 1 | Left, alive |
| 837 | alive | F | 40 | 13.3 | 108 | 18.1 | 74.6 | 7.4 | 15 | 1 | Left, alive |
| 846 | dead | F | * | 8.1 | 43 | 8.6 | 91.3 | 0.1 | 3 | 1 | Right, dead |
| 853 | dead | M | 80 | 12.3 | 75 | 5.9 | 89.3 | 4.9 | 9 | 0 | Vertical, dead |
| 857 | dead | F | * | 15.6 | 9 | 10.9 | 82.2 | 6.9 | 3 | 1 | Left, dead |
| 909 | alive | F | * | 14.8 | 8 | 49.5 | 38.2 | 12.3 | 3 | 0 | Left, alive |
| 923 | alive | F | * | 13.4 | 98 | 13.0 | 78.2 | 8.8 | 3 | 1 | Left, alive |
| 933 | dead | M | * | 13.2 | 62 | 11.2 | 82.8 | 6.0 | 3 | 1 | Left, dead |
| 971 | alive | M | * | 14.2 | * | 5.3 | 91.3 | 3.4 | 3 | 1 | Vertical, alive |
| 972 | dead | M | 77 | 6.8 | * | 10.3 | 74.7 | 15.0 | 3 | 1 | Left, dead |
| 985 | dead | M | * | 15.4 | 131 | 7.8 | 79.9 | 12.2 | 3 | 1 | Left, dead |
| 993 | alive | M | * | 8.5 | * | 29.1 | 58.8 | 12.1 | 3 | 1 | Left, alive |
| 999 | alive | M | * | 13.5 | * | 6.4 | 89.5 | 4.2 | 3 | 1 | Vertical, alive |
| 2166 | dead | F | * | 11.9 | 21 | 10.4 | 76.9 | 12.7 | 3 | 1 | Left, dead |
| 2755 | alive | M | 80 | 10.3 | 45 | 29.9 | 57.1 | 12.9 | 14 | 1 | Left, alive |
| 5677 | alive | M | * | 7.2 | 174 | 18.5 | 72.3 | 9.2 | 1 | 1 | Left, alive |

- Spatial subsets were classified according to the patterns described in Figure 7b.
- The essential analyses conducted in this report can be reproduced:
  - - by plotting the continuous (L, M, or N) data provided in Tables S1 and S2, as well

as the discontinuous biological conditions (‘dead’/’alive’), the plots reported in Figs.

1a-d can be reproduced, documenting that the conditions cannot be distinguished;

- - - using the discrete sub-class assigned to each observation (‘left’, ‘vertical’, or ‘right’,

which are further subdivided into ‘alive’ and ‘dead’), readers can confirm that some

data subsets (reported in Figs. 9a, b) differed and achieved statistical significance;

- - - together, these two analyses demonstrate that blood leukocyte data possess some

hidden interactions (not observed in the first analysis), which can be deduced when

comparisons among subsets reach statistically significant differences;

- - - similarly, the values and Chi square test results shown in Table 1 can be reproduced.

**Glossary of the terms reported in tables S1, 2**

* Outc.: outcome

* Sex: F (female), M (male)

* CRP (C-reactive protein)

* WBC (white blood cell count [thousands/ml])

* N, M, L (neutrophil, monocyte, lymphocyte relative percentages [for simplicity,

other leukocytes have been ignored])

* MS (microbial species isolated)

1. *Acinetobacter baumannii*

2. *Bacillus circulans*

3. Coagulase-negative staphylococci (CNS)

4. *Enterobacter cloacae*

5. *Escherichia coli*

6. *Enterococus faecalis*

7. *Enterococus faecium* (Vancomycin Resistant, VRE)

8. *Klebsiella pneumoniae*

9. 324 *Klebsiella pneumoniae*

10. *Propionibacterium acnes*

11. *Pseudomonas aeruginosa*

12. *Streptococcus mitis*

13. *Klebsiella oxytoca*

14. *Brucella spp*

15. *Staphylococcus epidermidis*

16. MRSA (methicilin-resistant *Staphylococcus aureus*)

17. *Candida parapsilosis*

18. *Staphylococcus hyicus*

19. *Stenotrophomonas moltophilia*

20. *Staphylococcu. aureus*

21. *Candida albicans*

22. *Klebsiella pneumoniae* (MBL)

23. *Serratia marcescens*

24. *E. mirabilis*

25. *S. liquefaciens*

* AB susc (susceptibility to antibiotics)-1: susceptible to ≥ 3 antibiotics; *: other

**Table S3. SIRS-negative, infection-negative study (n=20)**

| Case ID | Age | WBC | L% | N% | M% | Spatial subset* |
| --- | --- | --- | --- | --- | --- | --- |
| C16 | ≥ 21 | 4.2 | 6.3 | 57.4 | 36.1 | Other |
| C19 | ≥ 21 | 5.1 | 10.6 | 41.4 | 47.8 | Not inflamed |
| C9 | ≥ 21 | 6.7 | 8.1 | 63.2 | 28.5 | Other |
| C12 | ≥ 21 | 6.4 | 7.6 | 57.1 | 35.1 | Other |
| C8 | ≥ 21 | 5.9 | 8.6 | 65.5 | 25.8 | Other |
| C20 | ≥ 21 | 5.5 | 6.0 | 69.6 | 24.2 | inflamed |
| C3 | ≥ 21 | 7.8 | 5.1 | 51.0 | 43.8 | Other |
| C15 | ≥ 21 | 6.5 | 8.5 | 48.9 | 42.5 | Not inflamed |
| C11 | ≥ 21 | 8.0 | 8.8 | 66.6 | 24.4 | Other |
| C1 | ≥ 21 | 6.0 | 7.1 | 56.1 | 36.7 | Other |
| C10 | ≥ 21 | 8.6 | 7.2 | 61.8 | 30.9 | Other |
| C6 | ≥ 21 | 5.7 | 5.0 | 51.5 | 43.4 | Other |
| C14 | ≥ 21 | 7.2 | 5.0 | 69.6 | 25.2 | inflamed |
| C2 | ≥ 21 | 9.7 | 6.2 | 71.8 | 21.8 | inflamed |
| C18 | ≥ 21 | 6.2 | 7.2 | 73.1 | 19.5 | inflamed |
| C4 | ≥ 21 | 7.3 | 6.1 | 58.7 | 35.0 | Other |
| C13 | ≥ 21 | 7.6 | 10.3 | 60.8 | 28.8 | Other |
| C5 | ≥ 21 | 5.0 | 7.2 | 58.7 | 34.0 | Other |
| C17 | ≥ 21 | 7.1 | 9.3 | 52.0 | 38.5 | Not inflamed |
| C7 | ≥ 21 | 7.3 | 8.1 | 72.4 | 19.3 | Inflamed |

* Spatial subsets were classified according to the patterns described in Figure 6c.

**Table S4. SIRS+, infection+, longitudinal study (n=7)**

| Case  ID | Day | Outcome | Sex | Age | WBC | L% | N% | M% | MS | AB res | Spatial subset* |
| --- | --- | --- | --- | --- | --- | --- | --- | --- | --- | --- | --- |
| 1 | 1 | dead | F | 92 | 41.2 | 2.4 | 96.6 | 1.0 | 5 | 1 | vertical |
| 1 | 2 | dead | F | 92 | 38.9 | 3.0 | 96.7 | 0.2 | * | 1 | right |
| 1 | 3 | dead | F | 92 | 44.2 | 3.6 | 94.7 | 1.7 | * | 1 | vertical |
| 2 | 1 | dead | M | 64 | 16.6 | 5.6 | 86.5 | 7.8 | 5 | 1 | left |
| 2 | 2 | dead | M | 64 | 13.0 | 13.4 | 73.8 | 12.6 | * | 1 | left |
| 2 | 3 | dead | M | 64 | 7.3 | 16.9 | 65.9 | 17.0 | * | 1 | left |
| 3 | 1 | dead | F | 76 | 15.5 | 18.2 | 73.6 | 8.0 | 6 | 1 | left |
| 3 | 2 | dead | F | 76 | 12.7 | 14.3 | 78.2 | 7.4 | * | 1 | left |
| 3 | 3 | dead | F | 76 | 24.7 | 6.6 | 87.9 | 5.4 | * | 1 | vertical |
| 4 | 1 | dead | M | 61 | 6.6 | 10.8 | 87.8 | 1.3 | 1 | 1 | vertical |
| 4 | 2 | dead | M | 61 | 4.0 | 7.6 | 92.2 | 0.1 | * | 1 | right |
| 4 | 3 | dead | M | 61 | 5.5 | 15.5 | 81.0 | 3.3 | * | 1 | vertical |
| 5 | 1 | alive | M | 66 | 8.5 | 8.4 | 87.8 | 3.7 | 24 | 1 | vertical |
| 5 | 2 | alive | M | 66 | 9.4 | 13.5 | 81.7 | 4.6 | * | 1 | left |
| 6 | 1 | dead | M | 84 | 8.9 | 10.0 | 88.1 | 1.8 | 6 | 1 | vertical |
| 6 | 2 | dead | M | 84 | 3.3 | 47.2 | 51.2 | 1.5 | * | 1 | left |
| 7 | 1 | dead | M | 84 | 10.6 | 11.2 | 76.5 | 12.1 | 25 | 1 | left |
| 7 | 2 | dead | M | 88 | 21.3 | 2.9 | 94.4 | 2.6 | * | 1 | vertical |
| 7 | 3 | dead | M | 88 | 18.3 | 3.4 | 92.2 | 4.3 | * | 1 | vertical |

- Spatial subsets were classified according to the patterns described in Figure 7d.
- While no Chi square test could be conducted because all cells would have four or less observations (including a zero value [no survivors] in the right/vertical subset), mortality differed between the right/vertical and left subsets: it was 100% in the right/vertical subset (4 deaths over 4 observations) and 66% (2 deaths over 3 observations) in the left subset.

**Table S5. Subset-specific statistical analyses ‒SIRS-negative, infection-negative, cross-sectional study (n=20)**

| **Comparisons between subsets (Mann-Whitney test for the median)** | ***P* value** |
| --- | --- |
| **Whole blood cell counts** |
| Inflamed vs. non-inflamed | >0.05 |
| Non-inflamed vs. other | >0.05 |
| Inflamed vs. other | >0.05 |
|  |  |
| **Percentages or ratios**  **Inflamed vs. non-inflamed** |  |
| Lymphocyte % | **<0.04** |
| Neutrophil % | **<0.04** |
| Monocyte % | **<0.04** |
| Phagocyte / lymphocyte ratio | **<0.04** |
| Mononuclear cell / neutrophil ratio | **<0.04** |
|  |  |
| **Non-inflamed vs. other** |  |
| Monocyte / neutrophil | **<0.03** |
| Mononuclear cell / N | **<0.03** |
| Phagocyte / L | =0.05 |
|  |  |
| **Inflamed vs. other** |  |
| Lymphocyte % | >0.05 |
| Neutrophil % | **<0.01** |
| Monocyte % | **<0.01** |
| Monocyte / neutrophil | **<0.01** |

**Table S6. Subset-specific statistical analyses ‒SIRS-positive, infection-positive, longitudinal study (n=8)**

| **Comparisons between subsets** (**Mann-Whitney test for the median)** | ***P* value** |
| --- | --- |
| **Temporal** |
| N, L, or M% day 1 vs. day 2 | >0.05 |
| N, L, or M% day 1 vs. day 3 | >0.05 |
| N, L, or M% day 2 vs. day 3 | >0.05 |
|  |  |
| **Spatial subsets** |  |
| N % (left, n=8) vs. N % (vertical, n=9) | **< 0.01** |
| L % (left, n=8) vs. L % (vertical, n=9) | **=0.01** |
| M % (left, n=8) vs. NM % (vertical, n=9) | **< 0.01** |
| N % (left, n=8) vs. N % (right, n=2) | =005 |
| L % (left, n=8) vs. L % (right, n=2) | >005 |
| M % (left, n=8) vs. M % (right, n=2) | =005 |
|  |  |

**Table S7. Subset-specific statistical analyses ‒study I (n=36)**

| **Comparisons within and between subsets (Mann-Whitney test for the median)** | ***P* value** |
| --- | --- |
| **A- Comparisons within survivors (data structure I)** |
| i.- Lymphocyte % (left, alive, n=16) vs. lymphocyte % (vertical, alive, n=2) | **< 0.03** |
| ii.- Lymphocyte % (left, alive, n=16) vs. lymphocyte % (right, alive, n=2) | **< 0.03** |
| iii.- Neutrophil % (left, alive, n=16) vs. neutrophil % (vertical, alive, n=2) | **< 0.03** |
| iv.- Neutrophil % (left, alive, n=16) vs. neutrophil % (right, alive, n=2) | **< 0.03** |
| v.- Monocyte % (left, alive, n=16) vs. monocyte % (vertical, alive, n=2) | =0.058 |
| vi.- Monocyte % (left, alive, n=16) vs. monocyte % (right, alive, n=2) | **< 0.03** |
| **B- Comparisons within survivors (data structure II)** |  |
| i.- Lymphocyte % (left, alive, n=3) vs. lymphocyte % (vertical, alive, n=13) | **< 0.02** |
| ii.- Lymphocyte % (left, alive, n=3) vs. lymphocyte % (right, alive, n=4) | =0.052 |
| iii- Neutrophil % (left, alive, n=3) vs. neutrophil % (vertical, alive, n=13) | **< 0.02** |
| iv.- Neutrophil % (left, alive, n=3) vs. neutrophil % (right, alive, n=4) | =0.052 |
| v- Monocyte % (left, alive, n=3) vs. monocyte % (vertical, alive, n=13) | **< 0.04** |
| vi.- Monocyte % (left, alive, n=3) vs. monocyte % (right, alive, n=4) | =0.052 |
| vii.- CRP (left, alive, n=2) vs. CRP (vertical, alive, n=7) | > 0.10 |
| **C- Comparisons within non-survivors (data structure I)** |  |
| i.- Lymphocyte % (left, dead, n=9) vs. lymphocyte % (vertical, dead, n=6) | **< 0.01** |
| ii.- Neutrophil % (left, dead, n=9) vs. neutrophil % (vertical, dead, n=6) | **< 0.01** |
| iii.- Monocyte % (left, dead, n=9) vs. monocyte % (vertical, dead, n=6) | **< 0.03** |
| iv.- CRP (left, dead, n=5) vs. CRP (vertical, dead, n=3) | > 0.10 |
| **D**- **Comparisons within non-survivors (data structure II)** |  |
| i.- Lymphocyte % (lower left, dead, n=4) vs. lymphocyte % (vertical, dead, n=6) | **< 0.02** |
| ii.- Lymphocyte % (upper left, dead, n=3) vs. lymphocyte % (vertical, dead, n=6) | **< 0.03** |
| iii.- Lymphocyte % (vertical, dead, n=6) vs. lymphocyte % (right dead, n=3) | **< 0.03** |
| iv.- Neutrophil % (lower left, dead, n=4) vs. neutrophil % (upper left, dead, n=3) | =0.052 |
| v.- Neutrophil % (lower left, dead, n=4) vs. neutrophil % (vertical, dead, n=6) | **< 0.02** |
| vi- Neutrophil % (upper left, dead, n=3) vs. neutrophil % (vertical, dead, n=6) | **< 0.03** |
| vii.- Neutrophil % (vertical, dead, n=6) vs. neutrophil % (right dead, n=3) | **< 0.03** |
| viii.- Monocyte % (lower left, dead, n=4) vs. monocyte % (upper left, dead, n=3) | =0.052 |
| ix.- Monocyte % (lower left, dead, n=4) vs. monocyte % (vertical, dead, n=6) | **< 0.02** |
| x.- Monocyte % (lower left, dead, n=4) vs. monocyte % (right, dead, n=3) | =0.052 |
| xi.- CRP (lower left, dead, n=3) vs. CRP (vertical, dead, n=3) | >0.10 |
| **E- Comparisons between survivors and non-survivors (data structure I)** |  |
| i.- Lymphocyte % (left, alive, n=16) vs. lymphocyte % (vertical, dead, n=6) | **< 0.01** |
| ii.- Neutrophil % (left, alive, n=16) vs. neutrophil % (vertical, dead, n=6) | **< 0.01** |
| iii.- Monocyte % set 1 (left, dead , n=9) vs. monocyte % (right, alive, n=2) | **< 0.05** |
| **F- Comparisons between survivors and non-survivors (data structure II)** |  |
| i.- Lymphocyte % (lower left, alive, n=3) vs. lymphocyte % (vertical, dead, n=6) | **< 0.02** |
| ii.- Lymphocyte % (lower left, dead, n=4) vs. lymphocyte % (right, alive, n=4) | **< 0.04** |
| iii.- Neutrophil % (lower left, alive, n=3) vs. neutrophil % (vertical, dead, n=6) | **< 0.01** |
| iv.- Neutrophil % (lower left, dead, n=4) vs. neutrophil % (right, alive, n=4) | **< 0.04** |
| v. Neutrophil % (right, alive, n=4) vs. neutrophil % (right, dead, n=3) | =0.052 |
| vi. Monocyte % (lower left, alive, n=3 vs. monocyte % (vertical, dead, n=6) | **< 0.03** |
| vii. Monocyte % (lower left, dead, n=4 vs. monocyte % (right, alive, n=4) | **< 0.04** |

**Table S8. Subset-specific statistical analyses –study II (n=69)**

| **Comparisons within and between subsets (Mann-Whitney test for the median)** | ***P* value** |
| --- | --- |
| **A- Comparisons within survivors (data structure I)** |
| i.- Lymphocyte % (left, alive, n=27) vs. lymphocyte % (vertical, alive, n=10) | **< 0.01** |
| ii.- Lymphocyte % (left, alive, n=27) vs. lymphocyte % (right, alive, n=3) | **< 0.02** |
| iii.- Neutrophil % (left, alive, n=27) vs. neutrophil % (vertical, alive, n=10) | **< 0.01** |
| iv.- Neutrophil % (left, alive, n=27) vs. neutrophil % (right, alive, n=3) | **<0.01** |
| v.- Monocyte % (left, alive, n=27) vs. monocyte % (vertical, alive, n=10) | =0.09 |
| **B- Comparisons within survivors (data structure II)** |  |
| i.- Lymphocyte % (left, alive, n=28) vs. lymphocyte % (vertical, alive, n=9) | **< 0.01** |
| ii.- Lymphocyte % (left, alive, n=28) vs. lymphocyte % (right, alive, n=3) | **< 0.01** |
| iii- Neutrophil % (left, alive, n=28) vs. neutrophil % (vertical, alive, n=9) | **< 0.01** |
| iii- Neutrophil % (left, alive, n=28) vs. neutrophil % (right, alive, n=3) | **< 0.01** |
| v- Monocyte % (left, alive, n=28) vs. mon ocyte % (right, alive, n=3) | **< 0.01** |
| **C- Comparisons within non-survivors (data structure I)** |  |
| i.- Lymphocyte % (left, dead, n=28) vs. lymphocyte % (vertical, dead, n=3) | **< 0.01** |
| ii.- Neutrophil % (left, dead, n=7) vs. neutrophil % (vertical, dead, n=20) | **< 0.01** |
| iii.- Monocyte % (left, dead, n=7) vs. monocyte % (vertical, dead, n=20) | **< 0.01** |
| iv.- CRP (left, dead, n=4) vs. CRP (vertical, dead, n=12) | >0.10 |
| **D**- **Comparisons within non-survivors (data structure II)** |  |
| i.- Lymphocyte % (left, dead, n=7) vs. lymphocyte % (vertical, dead, n=20) | **< 0.01** |
| ii.- Neutrophil % (left, dead, n=7) vs. neutrophil % (vertical, dead, n=20) | **< 0.01** |
| iii.- Monocyte % (left, dead, n=7) vs. monocyte % (vertical, dead, n=20) | **< 0.01** |
| iv.- Lymphocyte % (left, dead, n=7) vs. lymphocyte % (right, dead, n=2) | =0.057 |
| v.- Neutrophil % (left, dead, n=7) vs. neutrophil % (right, dead, n=2) | =0.057 |
| vi.- Monocyte % (left, dead, n=7) vs. monocyte % (right, dead, n=2) | =0.057 |
| vii.- Lymphocyte % (vertical, dead, n=20) vs. lymphocyte % (right, dead, n=2) | =0.077 |
| viii.- Neutrophil % (vertical, dead, n=20) vs. neutrophil % (right, dead, n=2) | >0.10 |
| ix.- Monocyte % (vertical, dead, n=20) vs. monocyte % (right, dead, n=2) | **< 0.03** |
| x- CRP (left, dead, n=4) vs. CRP (vertical, dead, n=12) | >0.10 |
| xi.- CRP (vertical, dead, n=12) vs. CRP (right, dead, n=2) | >0.10 |
| **E- Comparisons between survivors and non-survivors (data structure I)** |  |
| i.- Lymphocyte % (left, alive, n=27) vs. lymphocyte % (left, dead, n=8) | >0.10 |
| ii.- Neutrophil % (left, alive, n=27) vs. neutrophil % (left, dead, n=8) | >0.10 |
| iii.- Monocyte % (left, alive, n=27) vs. monocyte % (left, dead, n=8) | =0.067 |
| iv. CRP (left, alive, n=17) vs. CRP (left, dead, n=6) | >0.10 |
| **F- Comparisons between survivors and non-survivors (data structure II)** |  |
| i.- Lymphocyte % (left, alive, n=28) vs. lymphocyte % (left, dead, n=7) | >0.10 |
| ii.- Neutrophil % (left, alive, n=28) vs. neutrophil % (left, dead, n=7) | >0.10 |
| iii. Monocyte % (left, alive, n=28 vs. monocyte % (left, dead, n=7) | >0.10 |
| iv. CRP (left, alive, n=18) vs. CRP (left, dead, n=4) | >0.10 |
| i.- Lymphocyte % (left, alive, n=3) vs. lymphocyte % (left, dead, n=2) | >0.10 |
| ii.- Neutrophil % (vertical, alive, n=3) vs. neutrophil % (vertical, dead, n=2) | >0.10 |
| iii. Monocyte % (vertical, alive, n=3 vs. monocyte % (vertical, dead, n=2) | >0.10 |

**Figure S1. Three-dimensional patterns of antimicrobial resistance data.** No particular antibiotic seemed to explain the two perpendicular data subsets. For instance, in both study I (**a**) and II (**b**), most isolates were sensitive to three or more antibiotics.

**Fig. S1**

**A**

**B**

**Figure S2. Three-dimensional patterns of microbial test results.** No particular bacterial species seemed to explain the two perpendicular data subsets. For instance, in both study I (**a**) and II (**b**), coagulase-negative staphylococci (CNS) were found in both subsets and three of more bacterial species were detected in each subset.

**A**

**Fig. S2**

**B**

**Figure S3. Three-dimensional patterns of SIRS-negative, non-infected individuals.** Two partially different data structures (**a, b**) revealed three non-overlapping data clusters. The hypothesis that the three subsets differed in immune profiles was supported: subset A (**a, b**) was labeled ‘inflamed’ because it revealed higher neutrophil percentages and lower monocyte percentages than the remaining subsets (red line, **c**); subset B (**a, b**) was labeled ‘non-inflamed’ because it showed higher lymphocyte and lower neutrophil percentages than the ‘inflamed’ subset (**c**); while subset C (**a, b**) was assigned ‘other’ by default (**c**).

**Fig. S3**

**Figure S4. Three-dimensional patterns of gender data.** No gender explained the two perpendicular data subsets. In both study I (**a**) and II (**b**), observations from both genders were found in each subset.

**A**

**Fig. S4**

**B**
